# Neutrophil‐Mimetic MRI Enables Ultra‐Early Detection of Vascular Inflammation After Stroke

**DOI:** 10.1002/adhm.71325

**Published:** 2026-06-08

**Authors:** Marion Isabelle Morvan, Damien Levard, Eloïse Lemarchand, Amal Boublay, Charly Helaine, Jolan Malherbe, Mikaël Naveau, Didier Goux, Igor Khalin, Ankita Talukdar, Cheun Pelleter, Charlène Jacqmarcq, Erwan Baudron, Isabelle Bardou, Sara Martinez de Lizarrondo, Maxime Gauberti, Denis Vivien, Antoine Philippe Fournier

**Affiliations:** ^1^ Institut Blood and Brain @ Caen‐Normandie (BB@C) INSERM UMR‐S U1237 Physiopathology and Imaging of Neurological Disorders (PhIND) GIP Cyceron UNICAEN, Université Caen Normandie Normandie University Caen France; ^2^ UAR 3408/US 50 Cyceron, CNRS, INSERM GIP CYCERON University of Caen Normandy Normandie Université Caen France; ^3^ UNICAEN CMAbio3: Centre de Microscopie Appliquée à la Biologie US Emerode Normandie University Caen France; ^4^ Institute For Stroke and Dementia Research (ISD) LMU University Hospital Munich Germany; ^5^ Department of Diagnostic Imaging and Interventional Radiology Centre Hospitalier Universitaire Caen Caen France; ^6^ Department of Clinical Research Caen‐Normandie University Hospital, CHU Caen France

**Keywords:** cell adhesion molecule, endothelial cell, molecular MRI, neuroinflammation, neutrophil, stroke

## Abstract

Following ischemic stroke, neutrophil adhesion to activated cerebral endothelium triggers the initial inflammatory cascade. Real‐time, quantitative in vivo detection of this early interaction could help identify individuals most likely to benefit from immunomodulatory therapies. To achieve this, we analyzed single‐cell RNA sequencing data from brain tissue and identified E‐selectin (*Sele*) as one of the earliest adhesion molecules selectively upregulated in activated endothelial cells with a venous‐like transcriptional signature. We then developed iron oxide microparticles targeting E‐selectin, designed to mimic neutrophil adhesion and to function as MRI probes for early endothelial activation. Within seconds of injection, these probes adhered to inflamed vessels in models of LPS‐induced neuroinflammation and ischemic stroke, allowing rapid emergency imaging. We observed an MRI‐detectable signal as early as 4 h following LPS stimulation and 8 h post‐stroke. This binding was significantly associated with neutrophil infiltration, but not with lesion volume, blood‐brain barrier disruption, or the accumulation of T cells and monocyte‐derived cells. These findings demonstrate its specificity for neutrophil‐driven inflammation and its relevance as a biomarker of ultra‐early immune activation. These results suggest that neutrophil‐mimetic MRI probes could represent a promising approach for detecting the initial phase of stroke‐induced inflammation and for guiding personalized immunomodulatory strategies.

## Graphical abstract

In this study, we developed neutrophil‐mimetic MRI probes that detect ultra‐early neuroinflammation following ischemic stroke by targeting E‐selectin. Within seconds of their injection, these microparticles mimic initial leukocyte adhesion to the activated cerebral endothelium. This non‐invasive approach enables the unmasking of early neutrophil recruitment using MRI, providing a valuable strategy to guide personalized immunomodulatory therapies during the acute phase.

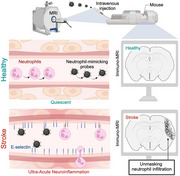

## 1 Introduction

Ischemic stroke is a primary cause of death and chronic functional impairment in adults and therefore represents a major global challenge. In most cases, it is caused by a thromboembolic occlusion of a cerebral artery, which deprives downstream brain tissue of oxygen and nutrients. Beyond this initial vascular event, a rapid and dynamic immune response unfolds. Increasing evidence has suggested that this response can contribute to the progression and outcome of stroke [1, 2]. Notably, the initial wave of immune infiltration in the ischemic brain is largely composed of neutrophils, which adhere to activated endothelial cells and initiate the early inflammatory cascade [3, 4].

Growing preclinical evidence has supported the therapeutic potential of immunomodulation in stroke [5, 6, 7]. However, translating these findings into clinical benefits has proven difficult, as most trials have failed to show significant efficacy [8, 9, 10, 11, 12]. One key limitation is the absence of tools for stratifying patients based on their inflammatory profile. Recent immunophenotyping data have revealed considerable variability in early immune responses, detectable as early as 3 h following symptom onset [13], suggesting that only a subset of patients may benefit from anti‐inflammatory interventions. Yet, current clinical imaging modalities are unable to visualize or quantify neuroinflammation within this ultra‐acute time frame.

In the era of precision medicine, there is a great unmet need for rapid and non‐invasive imaging techniques capable of detecting inflammation at its early stages. Immuno‐magnetic resonance imaging (immuno‐MRI) has emerged as a promising approach, using iron oxide microparticles conjugated to antibodies targeting endothelial adhesion molecules. This technique enables in vivo detection of inflammation‐induced vascular activation with high spatial resolution and without the use of ionizing radiation. Previous studies have demonstrated the value of immuno‐MRI in targeting vascular adhesion molecules such as VCAM‐1 [14, 15, 16, 17], ICAM‐1 [18, 19], MAdCAM‐1 [20, 21], MCAM [22], and P‐selectin [23, 24, 25]. These strategies have enabled the detection of post‐stroke inflammation between 24 h and several days after middle cerebral artery occlusion (MCAO). However, there is an unmet need for new approaches capable of unmasking vascular activation during the ultra‐early phase, within hours of stroke onset, when immune‐vascular interactions first emerge, and therapeutic opportunities are most viable.

To address this challenge, we aimed to develop an imaging strategy that mimics the behavior of neutrophils, the immune system's first responders to cerebral ischemia. To identify a vascular target expressed during this early window, we analyzed single‐cell RNA sequencing data from brain tissue and identified *Sele* (E‐selectin, CD62E) as a key adhesion molecule selectively upregulated following stroke in endothelial cells with a venous‐like transcriptional signature. We then developed superparamagnetic iron oxide microparticles conjugated to anti‐E‐selectin antibodies. These neutrophil‐mimicking probes were validated in vivo in both an LPS‐induced neuroinflammation model and a mouse model of ischemic stroke. Our findings demonstrate that these probes rapidly and specifically adhere to activated endothelium, enabling ultra‐early detection of cerebrovascular inflammation and revealing associated neutrophil infiltration by MRI.

## 2 Results

### 2.1 RNA Sequencing Identifies Selectins as Potential Markers of Early Endothelial Activation in a Model of Ischemic Stroke

**FIGURE 1:**
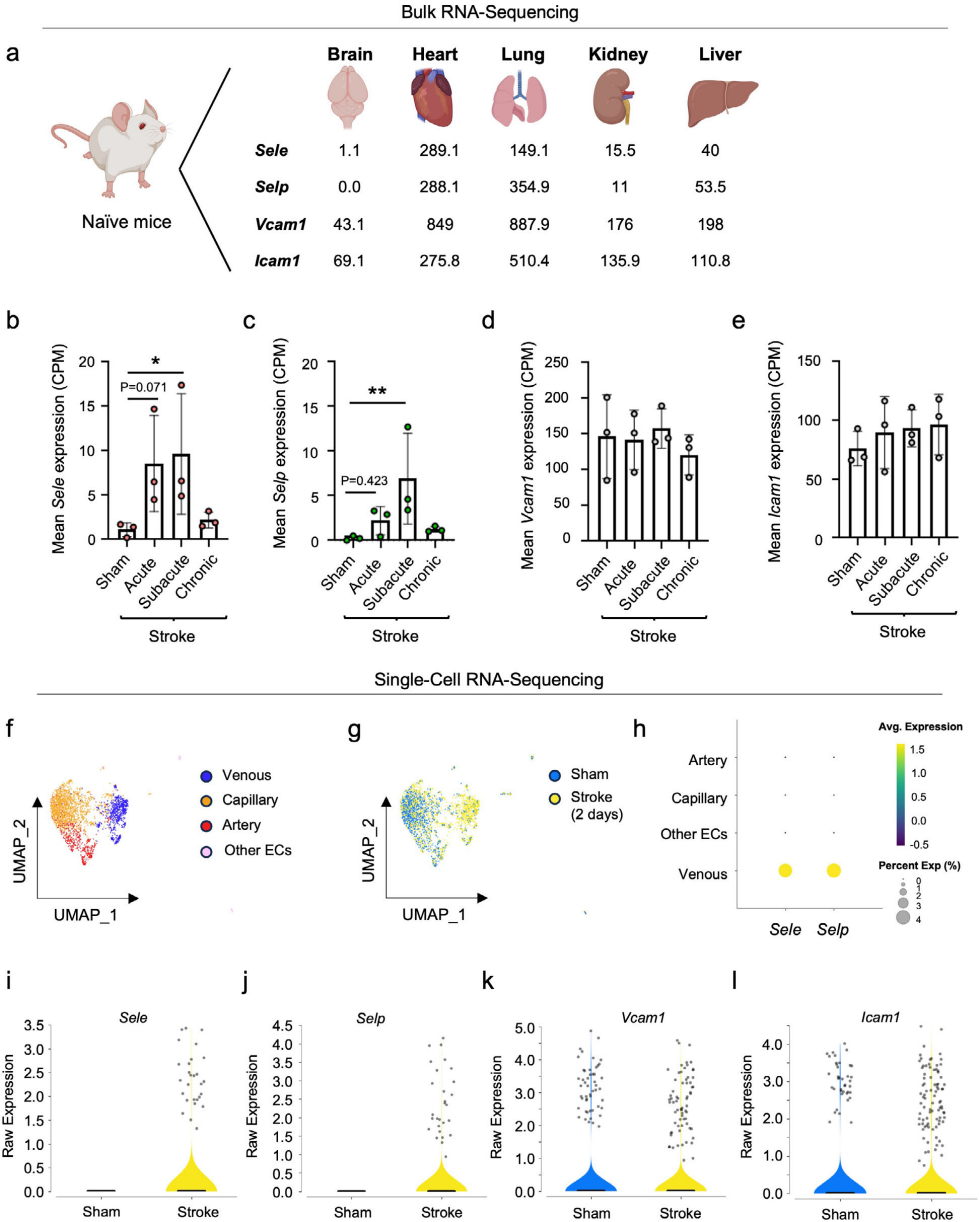
Transcriptomic analysis highlights selectins as early indicators of endothelial activation in ischemic stroke. (a–e) Bulk RNA sequencing dataset [26]. (a) *Sele*, *Selp*, *Vcam1*, and *Icam1* mRNA expression in endothelial cells from the brain, heart, lung, kidney, and liver of naïve mice (mean counts per million, CPM). Illustration created using BioRender.com. (b–e) *Sele*, *Selp*, *Vcam1*, and *Icam1* mRNA expression in brain endothelial cells from sham and stroke mice at different time points (mean CPM ± SD, *n =* 3). Statistical analyses were performed using the Kruskal–Wallis test + Dunn's multiple comparison test. ^*^ *p* < 0.05. ^**^ *p* < 0.01. Acute: 24 h post‐MCAO; subacute: 72 h; chronic: 1 month. (f–l) Single cell RNA sequencing dataset [27]. Data were obtained from cells isolated 2 days after MCAO. (f) Uniform manifold approximation and projection (UMAP) of brain endothelial cells colored by transcriptional populations. (g) UMAP of all cell clusters, colored by disease condition (sham vs stroke). (h) Dot plot showing the expression of *Sele* and *Selp* in endothelial cell populations. (i–l) Violin plot of *Sele, Selp, Vcam1*, and *Icam1* expression in endothelial cells from sham and stroke mice.

To identify adhesion molecules as markers of early endothelial activation in ischemic stroke, we analyzed publicly available RNA sequencing datasets from naïve mice and mice subjected to an ischemic stroke model [26, 27]. Bulk RNA sequencing analysis of endothelial cells isolated from various organs in naïve animals revealed a unique property of the brain vessels: the absence of *Sele* (encoding E‐selectin) and *Selp* (encoding P‐selectin) expression under healthy conditions (Figure 1a), whereas a basal expression of *Vcam1* and *Icam1* was detected. Moreover, our analysis demonstrated that *Sele* and *Selp* are significantly upregulated in brain endothelial cells during the subacute phase in a model of ischemic stroke (Figure 1b,c). No significant variations in the expression of *Vcam1* and *Icam1* were detected in these same mice (Figure 1d,e).

We included single‐cell RNA sequencing data in our analysis to resolve distinct endothelial cell populations (Figure 1f) in sham and stroke mice (Figure 1g). To identify these subtypes, we first isolated endothelial cells based on *Pecam1* and *Cldn5* expression, and then examined the expression of vascular bed markers previously identified in the literature (Figure S1a,b) [28, 29, 30]. Our analysis revealed that *Sele* and *Selp* expression was restricted to endothelial cells exhibiting a venous‐like transcriptional signature (Figure 1h) and was detected exclusively in stroke animals (Figure 1i,j). No expression of *Sele* and *Selp* was detected in sham animals, in contrast to other adhesion molecules like *Vcam1* and *Icam1*, which are expressed under basal conditions (Figure 1k,l). These findings highlight P‐selectin and E‐selectin as promising candidates for immuno‐MRI, due to their absence of expression in healthy conditions and their upregulation in stroke animals.

### 2.2 MPIO with a High Specificity for E‐Selectin Unmasks Neuroinflammation

**FIGURE 2:**
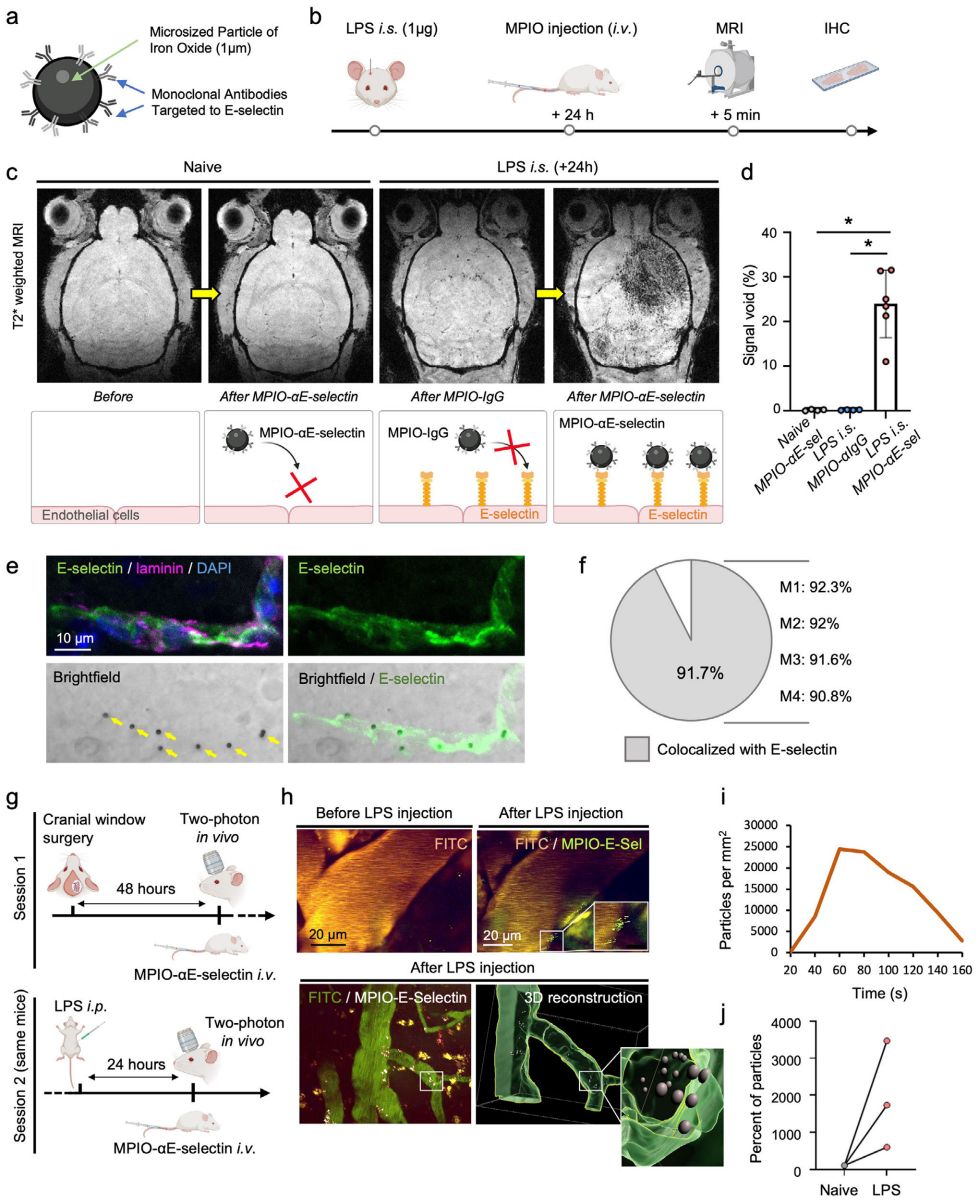
MPIO‐αE‐selectin selectively binds to activated endothelial cells in the murine brain. (a) Schematic representation of the structure of microsized particles of iron oxide (MPIO) conjugated with monoclonal anti‐E‐selectin antibodies. Illustration created using BioRender.com. (b) Schematic representation of the experimental procedure. *i.s*., intrastriatal; *i.v*., intravenous. Illustration created using BioRender.com. (c) Representative T2*‐weighted images after intravenous administration of MPIO targeted against E‐selectin or control MPIO‐IgG, 24 h after injection of 1 µg of LPS in the right striatum or in naive mice. (d) Corresponding quantification of MPIO‐αE‐selectin‐ and MPIO‐IgG‐induced signal voids (mean ± SD, *n =* 4–6). Statistical analyses were performed using the Kruskal–Wallis test + Dunn's multiple comparison test. ^*^ *p* < 0.05. E‐sel: E‐selectin. (e) Representative immunofluorescence image of MPIO‐αE‐selectin on the surface of an E‐selectin‐positive blood vessel in a mouse that received LPS 24 h before MPIO‐αE‐selectin injection. (f) Pie chart showing the proportion of MPIO‐αE‐selectin colocalized with E‐selectin immunosignal. M1‐4: mouse 1–4. (g) Schematic representation of the two‐photon microscopy experimental procedure. *i.p*., intraperitoneal; *i.v*., intravenous. Illustration created with BioRender.com. (h) Representative two‐photon images of brain blood vessels in a naïve mouse and 24 h after intraperitoneal LPS injection. MPIO‐αE‐selectin was injected at the start of image acquisition. (i) Kinetics of the number of visible particles in two‐photon imaging following intravenous injection in LPS‐treated mice. (j) Quantification of MPIO‐αE‐selectin binding in naïve mice and 24 h after LPS injection.

We previously developed microparticles of iron oxide (MPIOs) targeting P‐selectin for the detection of endothelial activation in models of transient ischemic attack and multiple sclerosis [23, 24]. To compare the efficacy of immuno‐MRI targeting P‐selectin and E‐selectin in the detection of acute endothelial activation, we next developed MPIOs targeting E‐selectin. For this, we used a monoclonal anti‐E‐selectin antibody (clone 9A9), which has been previously used in vivo to target E‐selectin [31, 32] (Figure 2a). The efficient coupling of monoclonal antibodies onto the MPIO surface was verified by flow cytometry (Figure S2) and confocal microscopy (Figure S3). Both approaches confirmed the presence of anti‐E‐selectin antibodies on the MPIOs designed to target E‐selectin (MPIO‐αE‐selectin), as demonstrated by the specific fluorescence signal generated upon incubation with fluorescent secondary antibodies. The mean hydrodynamic diameter of the MPIO‐αE‐selectin particles was 1090 nm, as measured by dynamic light scattering. The polydispersity index was 0.058, and the zeta potential at pH 7.4 was −12.7 mV (Figure S4). High‐resolution scanning electron microscopy (SEM) further confirmed the micrometer‐scale size and uniformity of the MPIOs in suspension (Figure S5). Using a 7T preclinical MRI system (300 MHz) at room temperature, the transverse (r_2_) and effective transverse (r_2_*) relaxivities of MPIO‐αE‐selectin were measured as 64.28 and 516.71 mm ^−^^1^ s^−^^1^, respectively, consistent with the strong superparamagnetic susceptibility effects of these particles. The longitudinal relaxivity (r_1_) was negligible under these conditions and could not be reliably quantified (Figure S6). To determine the specificity of MPIO‐αE‐selectin in vivo, we employed a neuroinflammation model induced by intrastriatal injection of *Escherichia coli* lipopolysaccharide (LPS) into the right striatum. MPIO injections and MRI acquisitions were performed 24 h post‐LPS injection (Figure 2b). Following intravenous injection of MPIO‐αE‐selectin (1 mg/kg of iron), numerous signal voids were detected in the right striatum using T2*‐weighted MRI (Figure 2c,d). However, no significant differences were detected between the right and left hemispheres after the injection of the same dose of control MPIO‐IgG in LPS‐treated mice. In naïve mice, no signal void was detected after the injection of the MPIO‐αE‐selectin (Figure 2c,d). After MRI acquisitions, brains from the LPS‐treated mice were isolated, and immunohistochemistry analysis confirmed, in accordance with MRI images, a vascular expression of E‐selectin (Figure S7b,c) and a binding of the particles only in the LPS‐injected striatum (Figure S7d,e). Our analysis also revealed that E‐selectin was mainly expressed in small vessels (mean diameter of 6.2 µm, Figure S7f). Because animals were transcardially perfused immediately after MRI, thereby removing any possible circulating MPIOs, the particles detected histologically correspond exclusively to vessel‐bound MPIO‐αE‐selectin. Quantification of these particles in perfused brain sections showed that more than 91% of the vessel‐bound MPIO‐αE‐selectin colocalized with E‐selectin expressed on the luminal surface, thereby confirming the high specificity of the particles (Figure 2e,f). Using intravital two‐photon microscopy, we investigated in vivo MPIO‐αE‐selectin binding before and 24 h after intraperitoneal LPS injection (Figure 2g,h). Due to the presence of a cranial window, LPS was administered intraperitoneally rather than intrastriatally to induce inflammation. A few minutes prior to MPIO‐αE‐selectin injection, FITC‐dextran (70 kDa) was injected intravenously to visualize the cerebral vasculature. We observed a peak in the number of circulating particles approximately 60 s post‐injection, followed by a rapid decrease. By 160 s, only particles bound to the blood vessel walls remained visible, reflecting a sub‐minute plasmatic half‐life (Figure 2i). Additionally, MPIO‐αE‐selectin particles were observed binding the luminal surface of cerebral blood vessels following LPS injection, with no evidence of extravasation across the vascular wall (Figure 2h,j).

**FIGURE 3:**
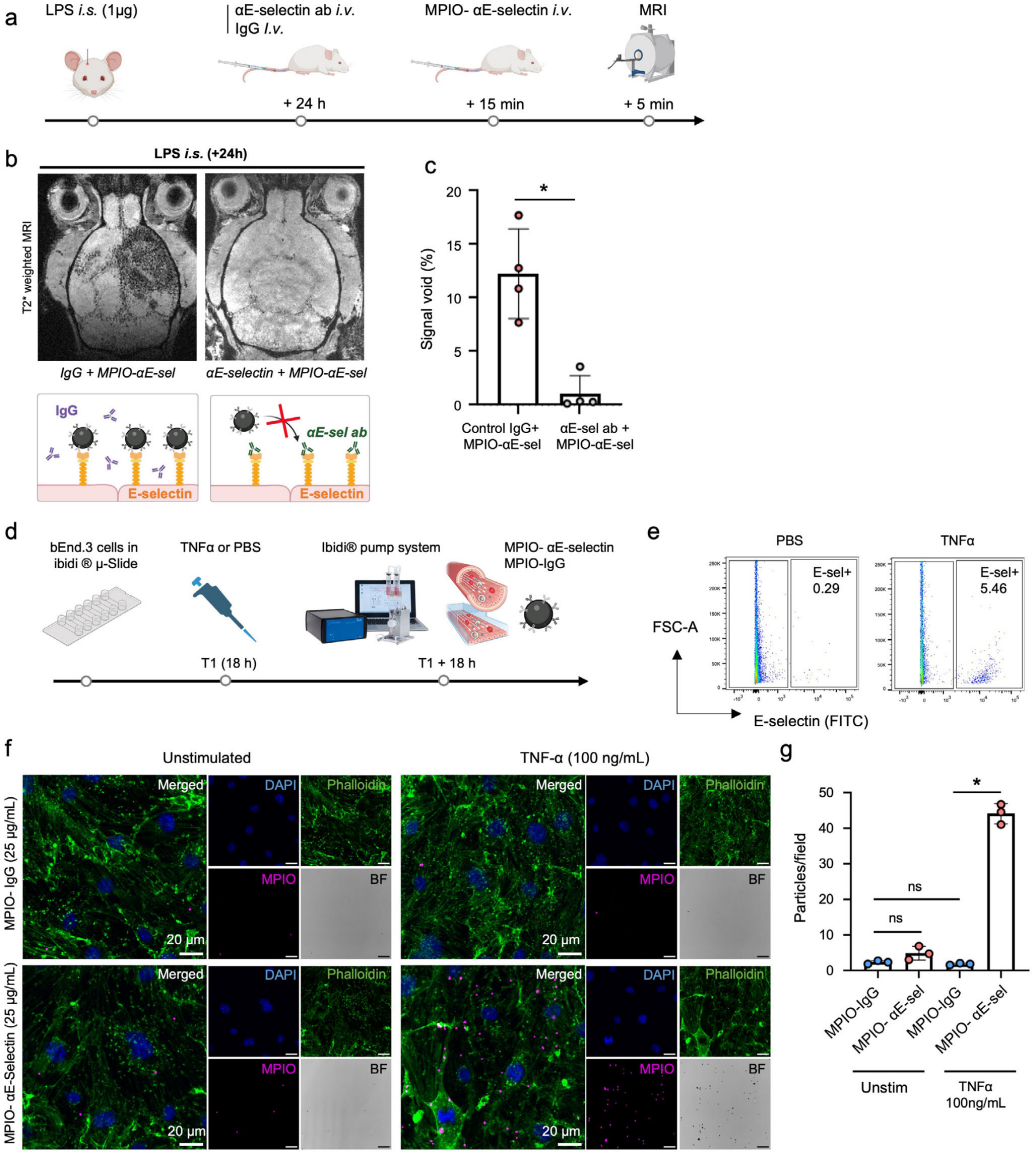
Demonstration of the specificity of MPIO‐αE‐selectin binding. (a) Schematic representation of the experimental procedure. *i.s*., intrastriatal; *i.v*., intravenous. (b) Representative T2*‐weighted images after intravenous administration of MPIO targeted against E‐selectin in mice treated with LPS and either antibodies against E‐selectin (αEsel ab) or control antibodies (control IgG), administered 15 min before MPIO‐αE‐selectin injection. (c) Corresponding quantification of MPIO‐αE‐selectin‐induced signal void (mean ± SD, *n =* 4). Statistical analyses were performed using Mann–Whitney U‐test. ^*^ *p* < 0.05. E‐sel: E‐selectin. (d) Schematic representation of the experimental procedure. (e) Representative flow cytometry dot plots showing the percentage of E‐selectin‐positive cells following stimulation with PBS or TNF‐α. (f) Representative immunofluorescence images of quiescent and activated (TNFα‐treated) bEnd.3 cells after 5 min of shear flow (4 dyn/cm^2^) with either MPIO‐ αE‐Selectin or MPIO‐ IgG particles. Scale bar = 20 µm. (g) Corresponding quantification of the number of MPIO‐αE‐selectin or MPIO‐IgG particles per field of 155 µm × 155 µm. *N* = 3 with 6 images per condition. Data are presented as mean ± SD. Statistical analyses were performed using Kruskal–Wallis test + Dunn's multiple comparison test. ^*^ *p* < 0.05.

To confirm that the MRI signal voids are due to the magnetic susceptibility effect of MPIO‐αE‐selectin bound to E‐selectin on activated endothelial cells, we conducted a competition experiment where mice were pretreated with a monoclonal anti‐E‐selectin antibody or control IgG (Figure 3a). This experiment demonstrated that saturating the binding sites before injection of MPIO‐αE‐selectin prevented signal changes on T2*‐weighted MRI images, confirming the specificity of the particles (Figure 3b,c). Repeated imaging 15 and 150 min after a single injection of the MPIO‐αE‐selectin revealed a detachment of the particles at 150 min, confirming their binding in the lumen of blood vessels and their absence of extravasation (Figure S8a–c). Biodistribution was quantified by magnetic particle imaging (MPI) at different time points after injection, revealing that approximately 2.5% of the injected dose bound transiently within the brain vasculature, while the remaining unbound particles showed fast accumulation in the liver (Figure S8d–f). These observations align with those obtained using in vivo two‐photon imaging, demonstrating a sub‐minute plasmatic half‐life of the circulating particles (Figure 2i). Ex vivo imaging confirmed preferential accumulation in the liver and spleen, consistent with uptake by the reticuloendothelial system, and no significant retention in other organs (Figure S8g–j).

Then, we assessed the binding of MPIO‐αE‐selectin and MPIO‐IgG in vitro. To this end, brain endothelial cells (bEnd.3) were cultured in Ibidi µ‐Slides and either left quiescent or activated with murine TNF‐α. The upregulation of E‐selectin expression following TNF‐α stimulation was confirmed by flow cytometry (Figure 3e). Both control and activated endothelial monolayers were subsequently connected to the Ibidi pump system to impose dynamic flow conditions (Figure 3d). After injection of either MPIO‐αE‐selectin or MPIO‐IgG, a unidirectional shear stress was applied for 5 min. MPIO‐αE‐selectin exhibited significantly greater binding to activated endothelial cells compared with control MPIO‐IgG (Figure 3f,g and Movie S1–S4). Furthermore, MPIO‐αE‐selectin bound markedly more to activated than to quiescent endothelial cells. Together, these findings demonstrate the specificity of MPIO‐αE‐selectin for activated endothelium.

### 2.3 MPIO‐αE‐Selectin Reveals Early Endothelial Activation Occurring During LPS‐Induced Neuroinflammation

**FIGURE 4:**
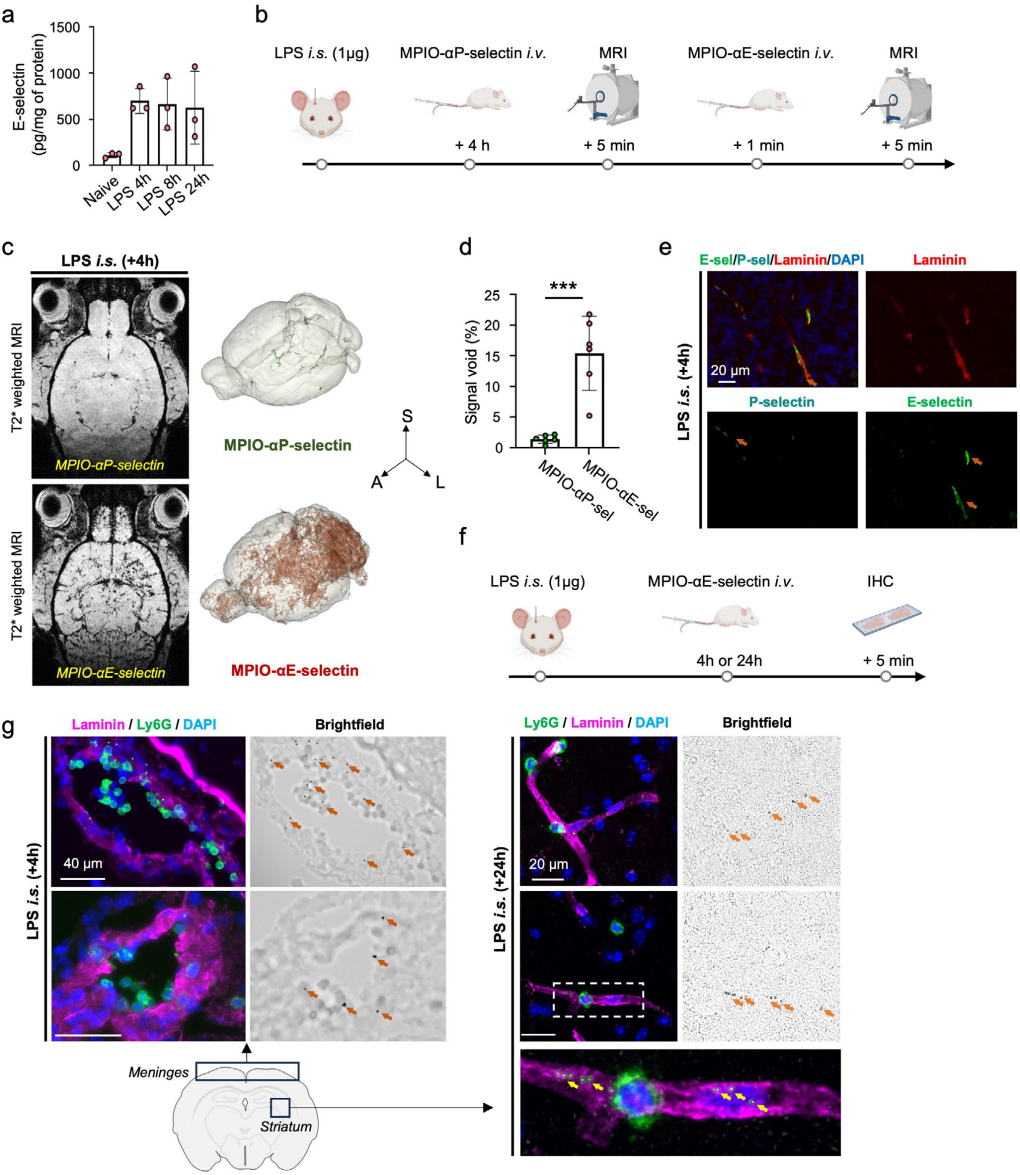
Immuno‐MRI targeting E‐selectin enables earlier detection of endothelial cell activation compared to P‐selectin targeting. (a) Quantification of E‐selectin expression by ELISA in brain tissue from naïve mice and from mice subjected to intrastriatal LPS injection (mean ± SD, n = 3 per group). (b) Schematic representation of the experimental procedure. *i.s*., intrastriatal; *i.v*., intravenous. Illustration created using BioRender.com. (c) Representative T2*‐weighted images after intravenous administration of MPIO targeted against E‐selectin or P‐selectin, 4 h after injection of 1 µg of LPS into the right striatum (left). Three‐dimensional reconstruction of MPIO‐induced signal void (right). (d) Corresponding quantification of MPIO‐induced signal void (mean ± SD, *n =* 6). Statistical analyses were performed using Student's *t*‐test. ^***^ *p* < 0.001. E‐sel: E‐selectin. P‐sel: P‐selectin. (e) Representative immunohistochemical images of E‐selectin and P‐selectin expression in cerebral blood vessels 4 h after LPS administration. (f) Schematic representation of the experimental procedure. *i.s*., intrastriatal; *i.v*., intravenous. Illustration created using BioRender.com. (g) Representative immunofluorescence images of MPIO‐αE‐selectin and Ly6G+ neutrophils in a mouse that received LPS 4 or 24 h before MPIO‐αE‐selectin injection.

Consistent with previous studies reporting leukocyte adhesion as early as 4 h after LPS administration [33], we observed an upregulation of E‐selectin expression in the brain beginning 4 h after intrastriatal LPS injection (Figure 4a). To assess the ability of MPIO‐αE‐selectin and MPIO‐αP‐selectin to detect early endothelial activation at this time point, T2*‐weighted MRI was performed sequentially in the same mice, first following injection of MPIO‐αP‐selectin, and then after injection of MPIO‐αE‐selectin (Figure 4b). This experiment demonstrated a significant signal void after the injection of MPIO‐αE‐selectin 4 h following LPS injection in the striatum. Moreover, we showed that the signal from the MPIO‐αE‐selectin was significantly higher than that from the MPIO‐αP‐selectin (Figure 4c,d), consistent with the results obtained by fluorescence microscopy (Figure 4e). Given that E‐selectin is involved in neutrophil activation and adhesion, and considering that neutrophils are known to be the first leukocytes to invade infected tissue, we assessed whether MPIO‐αE‐selectin binds to neutrophil adhesion sites. Using fluorescent microscopy, we demonstrated that MPIO‐αE‐selectin and neutrophils (Ly6G+) indeed bind to the endothelium at the same locations, illustrating the capacity of immuno‐MRI targeting E‐selectin to reveal early neutrophil adhesion to inflamed brain blood vessels in vivo (Figure 4f,g).

However, the co‐localization of MPIO‐αE‐selectin and neutrophils at the endothelial surface raises the question of potential steric competition for E‐selectin binding sites. To determine whether the presence of our contrast agent could hinder neutrophil recruitment, we quantified neutrophil infiltration into the brain parenchyma. Flow cytometry analysis revealed no significant difference in the frequency of infiltrated neutrophils (CD45^high^/Ly6G^+^) between mice injected with MPIO‐αE‐selectin and those receiving control MPIO‐IgG (Figure S9a–c). This lack of interference is likely attributable to the transient nature of MPIO binding, which occurs over a limited time window (Figure S8). This short duration minimizes potential competition, thereby preserving the natural progression of the inflammatory response.

### 2.4 MPIO‐αE‐Selectin Reveals Early Endothelial Activation Associated With Neutrophil Infiltration During Ischemic Stroke

**FIGURE 5:**
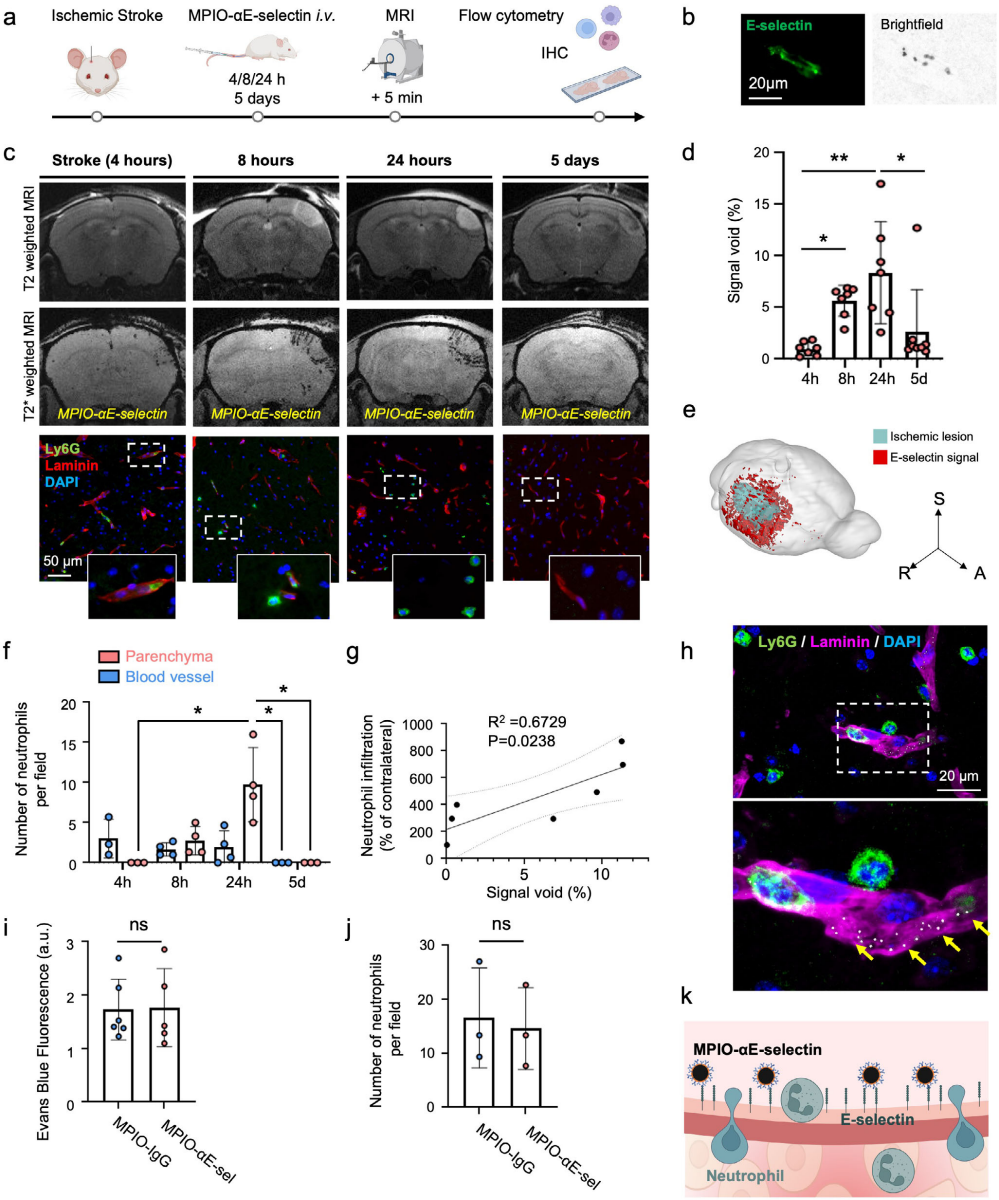
Immuno‐MRI targeting E‐selectin reveals early endothelial activation associated with neutrophil invasion in an ischemic stroke model. (a) Schematic representation of the experimental procedure. *i.v*., intravenous. Illustration created using BioRender.com. (b) Representative immunofluorescence images of MPIO‐αE‐selectin bound to the surface of an E‐selectin‐positive blood vessel acquired 24 h after MCAO induced by aluminum chloride. (c) Representative T2*‐weighted images obtained after intravenous administration of MPIO‐αE‐selectin alongside immunohistochemistry of Ly6G+ neutrophils performed 4, 8, 24 h, and 5 days post‐MCAO. (d) Quantification of MPIO‐αE‐selectin‐induced signal void in the right cortex (mean ± SD, *n =* 7–8). Statistical analysis was performed using the Kruskal‐Wallis Test + Dunn's multiple comparison test. ^*^ *p* < 0.05; ^**^ *p* < 0.01. (e) Three‐dimensional reconstruction of MPIO‐αE‐selectin‐induced signal void 24 h after stroke. (f) Quantification of neutrophil counts within blood vessels and infiltrated into the brain parenchyma (mean ± SD, *n =* 3–4 per group). Statistical analysis was performed using Kruskal–Wallis Test + Dunn's multiple comparison test. ^*^ *p* < 0.05. (g) Correlation between MPIO‐αE‐selectin‐induced signal void and neutrophil infiltration quantified by flow cytometry (*n =* 7). Reported statistics were obtained using Pearson's correlation test. (h) Representative immunofluorescence images of MPIO‐αE‐selectin and Ly6G+ neutrophils, acquired 24 h post‐MCAO. (i) Quantification of vascular permeability following Evans blue dye injection (mean ± SD, n = 5–6). Statistical analysis was performed using the Mann–Whitney U‐test. (j) Quantification of neutrophils counts by immunofluorescence microscopy (mean ± SD, *n =* 3 per group). Statistical analysis was performed using the Mann–Whitney U‐test. (k) Schematic representation illustrating the behavior of MPIO‐αE‐selectin, acting as neutrophil‐mimicking particles.

Neutrophils are known to be the first leukocytes to invade the brain after an ischemic stroke, typically within the first few hours following the occlusion [34]. To evaluate the capacity of immuno‐MRI targeting E‐selectin to unmask early neutrophil infiltration occurring during ischemic stroke, we performed an experiment where MPIO‐αE‐selectin was intravenously injected at 4, 8, 24 h, and 5 days after MCAO (Figure 5a). We demonstrated that MPIO‐αE‐selectin binds to E‐selectin‐positive vessels (Figure 5b), inducing a signal void that peaks at 8 and 24 h post‐MCAO. This signal was significantly reduced when particles were injected 5 days post‐MCAO (Figure 5c,d). Interestingly, MPIO‐αE‐selectin binding predominantly occurs at the periphery of the lesion, revealing the presence of an inflammatory penumbra (Figure 5e). In contrast, the signal observed after the injection of MPIO‐αP‐selectin was lower and did not show significant differences between the early time points (Figure S10a–c). Consistently, immunohistochemistry revealed a trend toward higher E‐selectin expression compared with P‐selectin during the early post‐stroke phase (Figure S10d,e). Using immunofluorescent microscopy, we confirmed that the expression of E‐selectin follows the same pattern as the MRI signal, with E‐selectin expression observed at 8 and 24 h (Figure S11a–c). Consistent with previous observations in the LPS model, we found that E‐selectin is predominantly expressed in small vessels in the ischemic stroke model (mean diameter of 6.5 µm, Figure S11d). Interestingly, we demonstrated that neutrophil infiltration begins at 8 h and reaches its peak at 24 h, aligning with the expression of E‐selectin and, consequently, the MRI signal from MPIO‐αE‐selectin (Figure 5f). To confirm these observations, we performed flow cytometry on mouse brains immediately following the MRI examinations to quantify the infiltration of various immune cell types (Figure S12). This experiment demonstrated a positive correlation between the MRI signal obtained from MPIO‐αE‐selectin and the magnitude of neutrophil recruitment in the brain (Figure 5g). In line with these observations, we revealed that MPIO‐αE‐selectin binds to the vascular wall at the same sites as neutrophil adhesion (Figure 5h,k, and Figure S13).

To determine whether MPIO‐αE‐selectin could compete with neutrophils for endothelial binding sites, we assessed neutrophil recruitment and vascular permeability following probe administration. Mice received either MPIO‐IgG or MPIO‐αE‐selectin at 8 h after MCAO, and Evans blue dye was injected at 24 h to evaluate blood‐brain barrier permeability by optical imaging. No difference in Evans blue extravasation was observed between groups (Figure 5i). Consistently, immunohistofluorescence quantification of infiltrating neutrophils revealed no detectable change associated with MPIO‐αE‐selectin injection (Figure 5j). These results indicate that MPIO‐αE‐selectin does not interfere with neutrophil recruitment during acute ischemic injury.

### 2.5 The Kinetics of E‐Selectin Expression Differ From Those of BBB Disruption and T Lymphocyte and Monocyte Invasion During Ischemic Stroke

**FIGURE 6:**
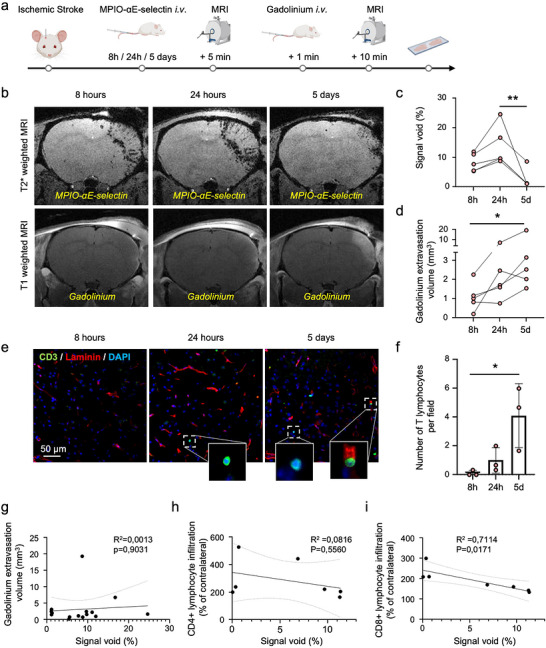
Immuno‐MRI targeting E‐selectin does not show correlation with blood‐brain barrier permeability or lymphocyte infiltration. (a) Schematic representation of the experimental procedure. *i.v*., intravenous. Illustration created using BioRender.com. (b) Representative T2*‐weighted images obtained after intravenous administration of MPIO‐αE‐selectin, alongside T1‐weighted images acquired after intravenous administration of gadolinium. (c) Quantification of MPIO‐αE‐selectin‐induced signal void in the right cortex (*n =* 5). Statistical analysis was performed using the Friedman multiple comparison test. ^**^ *p* < 0.01. (d) Quantification of gadolinium extravasation volume (*n =* 5). Statistical analysis was performed using the Friedman multiple comparison test. ^*^ *p* < 0.05. (e) Representative immunohistochemistry images of CD3+ lymphocytes at 8 and 24 h, and 5 days post‐MCAO. (f) Quantification of CD3+ lymphocytes in the brain parenchyma (mean ± SD, *n =* 3 mice per group). Statistical analysis was performed using the Kruskal–Wallis test + Dunn's multiple comparison test. ^*^ *p* < 0.05. (g) Correlation between MPIO‐αE‐selectin‐induced signal void and gadolinium extravasation volume. Reported statistics were obtained using Spearman's correlation test. (h) Correlation between MPIO‐αE‐selectin‐induced signal void and CD4+ lymphocyte infiltration quantified by flow cytometry (*n =* 7). Reported statistics were obtained using Spearman's correlation test. (i) Correlation between MPIO‐αE‐selectin‐induced signal void and CD8+ lymphocyte infiltration quantified by flow cytometry (*n =* 7). Reported statistics were obtained using Pearson's correlation test.

Having demonstrated that the MPIO‐αE‐selectin signal observed in MRI is positively correlated with neutrophil infiltration following ischemic stroke, our next step was to compare it with other neuroinflammatory events, such as increased blood‐brain barrier (BBB) permeability and T lymphocyte invasion. In the same mice, we performed a longitudinal experiment where E‐selectin expression was assessed using MPIO‐αE‐selectin (T2* weighted MRI) prior to the intravenous injection of gadolinium to assess BBB permeability (T1 weighted MRI) at 8 h, 24 h, and 5 days post‐MCAO (Figure 6a). We demonstrated that in contrast to the MPIO‐αE‐selectin‐associated MRI signal, which peaks at 8 and 24 h (Figure 6b,c), BBB permeability increases over time and is more pronounced at 5 days post‐MCAO (Figure 6b,d). Using immunohistochemistry, we showed that T lymphocyte invasion of the brain follows a similar kinetic to BBB disruption observed in MRI, namely an increase over time. Indeed, we observed that the number of CD3+ lymphocytes is significantly higher at 5 days than at 8 h (Figure 6e,f and Figure S12). In line with these observations, the E‐selectin signal showed no correlation with BBB permeability (Figure 6g). Additionally, flow cytometry analysis revealed no positive correlation between the MPIO‐αE‐selectin‐induced signal void and the infiltration of CD4+ or CD8+ lymphocytes (Figure 6h,i), nor with monocyte‐derived cell recruitment or lesion volume (Figure S14). These results demonstrate that, by binding to early‐expressed E‐selectin, MPIO‐αE‐selectin specifically mimic the behavior of neutrophils.

## 3 Discussion

In this study, we developed a neutrophil‐mimetic MRI probe that allows for rapid, sensitive, and non‐invasive detection of ultra‐early vascular activation following ischemic stroke. To mimic the adhesive behavior of neutrophils, which are the first immune cells to engage the activated endothelium, we designed E‐selectin‐targeted MPIOs that rapidly adhere to activated endothelial cells. The specificity of these probes was validated in vitro under physiological flow, where MPIO‐αE‐selectin demonstrated selective binding to activated brain endothelial cells, reflecting the TNF‐α‐induced E‐selectin upregulation confirmed by flow cytometry. We demonstrated their strong specificity for luminal E‐selectin in vivo and their rapid clearance after binding, supporting the suitability of this approach for repeated, longitudinal imaging. Compared to P‐selectin‐targeted counterparts, E‐selectin‐targeted MPIOs more effectively captured early endothelial activation. Interestingly, the MRI signal void induced by these probes correlated with the magnitude of neutrophil infiltration, but not with lesion volume, blood‐brain barrier permeability, or the accumulation of T cells and monocyte‐derived cells, underscoring their specificity for neutrophil‐driven inflammation. This strategy opens new perspectives for stratifying patients based on inflammatory status during the therapeutic window, and may ultimately support personalized immunomodulatory interventions in acute stroke.

Beyond its experimental relevance, this approach addresses one of the major obstacles to the clinical translation of immunomodulatory therapies in stroke: the lack of imaging tools to identify and stratify patients according to their inflammatory response. The ability to visualize cerebrovascular inflammation within hours of stroke onset could enable the early identification of individuals with excessive immune activation who are most likely to benefit from targeted interventions. E‐selectin‐targeted immuno‐MRI could thus offer a solution to the repeated failures of immunomodulatory clinical trials, in which patients were treated indiscriminately, without objective, imaging‐based evaluation of their inflammatory status.

Currently, only positron emission tomography (PET) is used for molecular imaging in humans. By imaging the translocator protein (TSPO) as a marker of glial cell reactivity, several studies reported PET's capacity to visualize neuroinflammation. However, this technique exhibits a poor signal‐to‐noise ratio, hindering the detection of small fluctuations in brain inflammation. In stroke, TSPO expression becomes elevated after several days, peaking at 3–4 weeks [35], although acute inflammation is known to occur within hours following ischemic stroke. Moreover, PET suffers from low spatial resolution, long acquisition times, and exposes both patients and medical staff to ionizing radiation.

The quantification of neuroinflammation biomarkers in cerebrospinal fluid has shown potential for detecting inflammation in the central nervous system during chronic diseases such as multiple sclerosis [36, 37, 38]. However, this approach causes discomfort for patients, requires time between sampling and results, lacks sensitivity and specificity, and is unable to localize brain inflammation. This approach is thus not suitable for acute conditions like stroke, which require rapid management.

Several clinical studies have shown increased soluble E‐selectin concentrations in the blood of patients with acute ischemic stroke, demonstrating endothelial activation and microvascular injury [39, 40, 41, 42]. Although these circulating biomarkers provide information on systemic inflammation, serum E‐selectin levels fail to detect endothelial expression at a given time point. Indeed, rather than reflecting active processes, these serum levels represent the cumulative shedding of E‐selectin that occurred previously at the cell surface, thus failing to capture the current level and spatial distribution of endothelial activation within the cerebrovascular network. Such constraints become particularly critical in the context of stroke, a condition characterized by highly localized endothelial activation that evolves dynamically over time. By enabling direct, non‐invasive visualization of E‐selectin on the luminal surface of brain vessels, the probes developed in our study overcome a critical gap in the assessment of acute neuroinflammation. The clinical relevance of this approach is reinforced by the fact that human‐derived models, such as human induced pluripotent stem cell‐derived endothelial cells (hiPSC‐ECs), also upregulate E‐selectin after stimulation. The fact that these inflammatory mechanisms are conserved between our murine model and human cells strengthens the clinical relevance of E‐selectin‐targeted imaging [43, 44].

Immuno‐MRI possesses good spatial resolution and sensitivity, making it a credible alternative for imaging stroke‐induced acute neuroinflammation. Previous studies have demonstrated the ability of immuno‐MRI to detect stroke‐induced brain inflammation between 1 and 21 days post‐occlusion, using MPIOs targeting VCAM‐1, ICAM‐1, or P‐selectin [17, 18, 24, 45]. According to our RNA sequencing analysis and prior studies showing that E‐selectin expression peaks earlier than other adhesion molecules [46, 47], targeting E‐selectin holds significant potential for detecting early inflammatory processes. In addition, brain endothelial cells are known to lack P‐selectin within their Weibel‐Palade bodies, unlike peripheral endothelia [48, 49]. This neurovascular specificity suggests that E‐selectin plays a predominant and early role in leukocyte recruitment within the CNS, further supporting its relevance as an imaging target in acute neuroinflammation.

In this study, we successfully developed anti‐E‐selectin MPIOs, enabling MRI detection of early endothelial activation occurring 8 h after MCAO. We demonstrated that E‐selectin expression detected in a stroke model using MRI is correlated with the infiltration of neutrophils, known to be among the first leukocytes to invade tissue following an injury. This observation is consistent with previous studies showing that E‐selectin is involved in inflammasome activation within neutrophils [50], as well as in their infiltration during stroke [51].

We obtained a stronger MRI signal with MPIO‐αE‐selectin than with MPIO‐αP‐selectin. This result can be explained by differences in the cellular distribution of their respective targets. E‐selectin expression is restricted to activated endothelial cells, ensuring that all circulating MPIO‐αE‐selectin remain available for vascular engagement. On the other hand, P‐selectin is also expressed by activated platelets and multiple immune cell subtypes, which can interact with MPIO‐αP‐selectin in the bloodstream and consequently reduce the pool of particles available for endothelial binding, leading to an attenuated MRI signal. Differences in antibody affinity may also contribute to this difference.

In this study, we used micro‐sized particles of iron oxide (MPIOs), which, compared to nanoparticles, have the advantage of carrying a larger amount of iron oxide, allowing for the binding of a greater quantity of contrast agent to the target. This enables rapid imaging after particle injection, as, unlike nanoparticles, there is no need to wait for a sufficient number of particles to bind to the target. Another reason allowing for quick image acquisition after injection is that circulating MPIOs, which do not bind to their target, have a short plasmatic half‐life of a few seconds [52], unlike nanoparticles that have a plasmatic half‐life of several hours. Moreover, due to their larger size, MPIOs cannot cross the vascular wall, even in cases of increased blood‐brain barrier permeability [23]. As a result, the MRI signal is exclusively due to particles binding to the luminal side of the blood vessel, rather than passive accumulation in the tissue.

Previous molecular imaging studies have targeted E‐selectin using gadolinium‐based contrast agents or ultrasmall superparamagnetic iron oxide nanoparticles [53, 54, 55, 56]. While these approaches successfully demonstrate the feasibility of E‐selectin imaging, they use contrast agents with long plasma half‐lives and potential accumulation in the brain parenchyma, complicating the signal interpretation. In contrast, the MPIO‐based approach used in our study is fundamentally different, as it enables rapid imaging after injection, restricts the MRI signal to luminally bound particles, and provides a highly specific readout of endothelial activation.

An essential factor for the in vivo use of micrometer‐sized contrast agents is their potential impact on microvascular circulation. In our study, the MPIO‐αE‐selectin exhibited a mean hydrodynamic diameter of 1.09 µm, which is substantially smaller than the typical lumen of mouse cerebral capillaries (approximately 5 µm) and the diameter of circulating erythrocytes (6–7 µm). Our intravital two‐photon microscopy observations demonstrated that these particles circulate freely within the microvasculature without inducing stagnant flow or capillary obstruction. This aligns with the absence of significant non‐specific MRI signal voids in naïve and MPIO‐IgG control groups, which would otherwise indicate mechanical entrapment. In addition, the high monodispersity of our formulation (PDI = 0.058) and the rapid clearance of unbound particles by the reticuloendothelial system prevent the risk of cumulative vascular occlusion.

While MPIO‐αE‐selectin particles bind to the same sites as neutrophils, our results show that they do not interfere with neutrophil infiltration. This lack of competition is due to the transient nature of MPIO binding, which occurs over a limited timeframe. Indeed, because unbound MPIOs are cleared from the circulation within seconds by the mononuclear phagocyte system, the window for potential competition with endogenous neutrophils remains extremely narrow. This short duration reduces potential competition for E‐selectin binding sites, thereby preserving the progression of the inflammatory response.

A limitation of our study is the use of non‐biodegradable MPIOs. However, we have previously reported the development of biocompatible MPIOs, which are composed of nanoparticle clusters that are degraded by the reticuloendothelial system [57]. The dose of iron used in MPIOs (1–4 mg/kg) is compatible with clinical use in humans, as the injection of up to 1000 mg of iron in the form of nanoparticles is clinically well tolerated and is assimilated into the endogenous iron stores without inducing any reported adverse effects. These elements, along with our study, support the future clinical translation of immuno‐MRI using neutrophil‐mimetic MRI probes for the early detection of stroke‐induced acute neuroinflammation.

## 4 Methods

### 4.1 Transcriptomic Analyses

The bulk RNA sequencing data used were derived from a published and publicly accessible dataset in which endothelial cells from various organs were isolated from naïve mice and mice subjected to an ischemic stroke model at different stages of the disease [26]. The single‐cell RNA sequencing data, obtained from the brain transcriptomes of control mice and mice with ischemic stroke (filament model, 2 days post‐MCAO, GSE225948) [27], were processed and analyzed using Trailmaker (https://app.trailmaker.parsebiosciences.com/; Parse Biosciences). Raw sequencing data were processed automatically, including the exclusion of dead or dying cells based on mitochondrial content filtering, with thresholds ranging from 1.2% to 9.32% per sample. Outliers in the gene‐to‐transcript distribution were detected and excluded using a linear regression model with prediction intervals set between 0.99965 and 0.99977. Cells with a high probability of being doublets were removed using the scDblFinder method, with probability thresholds ranging from 0.43 to 0.76. Data integration was performed using Seurat with Harmony batch correction, selecting 2000 highly variable genes and 30 principal components, which together explained 88.92% of the variance. Dimensionality reduction was conducted using Uniform Manifold Approximation and Projection (UMAP) with cosine distance, followed by Louvain clustering at a resolution of 0.8.

### 4.2 MPIO Functionalization and Characterization

Microparticles of iron oxide (MPIO; 1.08 µm diameter) with a p‐toluenesulfonyl‐reactive surface (Invitrogen) were used to target P‐ and E‐selectin. For this purpose, 100 µg of E‐selectin antibody (BioXcell BE0294), control mouse IgG (BioXcell BE0085), or P‐selectin antibody (R&D Systems, clone MAB737) were conjugated with MPIOs as previously described [58] in a borate buffer (pH 9.5) for 36 to 48 h at 37°C. To neutralize residual reactive groups, MPIOs were incubated for 24 h at 37°C in a blocking solution containing 0.5% BSA. MPIOs were then washed in PBS containing 0.1% BSA and sonicated before storage under agitation at 4°C. To validate antibody grafting, non‐functionalized MPIOs and MPIO‐αE‐selectin were incubated with a FITC‐conjugated anti‐rat secondary F(ab’)_2_ fragment at room temperature for 20 min, followed by a washing step. Data were acquired on a FACSVerse flow cytometer (Becton Dickinson, Franklin Lakes, NJ) and processed using FlowJo software version 10.10.0 (Becton Dickinson, Ashland, OR). We recorded 20 000 events per sample, focusing on the MPIO subpopulation defined by forward and side scatter profiles. The conjugation efficiency was determined by microvolume spectrophotometry (NanoPhotometer N50; Implen), showing that approximately 75 µg of IgG were successfully functionalized per 50 µL of MPIO, corresponding to a high surface density of nearly 60 000 antibodies per microparticle.

Physicochemical properties of the MPIO‐αE‐selectin, including hydrodynamic diameter, polydispersity index (PDI), and surface charge (zeta potential), were assessed by dynamic and electrophoretic light scattering using a Nano ZS apparatus (Malvern Instruments, Worcestershire, UK). Measurements were conducted at 25°C using a 633‐nm laser, with a fixed scattering angle of 173° for size determination.

### 4.3 Scanning Electron Microscopy (SEM)

MPIO were coated with platinum and characterized by a JSM‐7200F Scanning Electron Microscope (JEOL Europe SAS, Croissy‐sur‐Seine, France) at 3 kV.

### 4.4 MRI Relaxivity Measurements

MPIOs‐αE‐selectin were embedded in 2% agarose gels (Tris‐acetate‐EDTA buffer) across a range of iron concentrations spanning 0 to 0.71 mmol L^−^^1^. Samples were imaged at room temperature using a 7‐T PET‐MRI system (BioSpec, Bruker). The MRI protocol included: (i) determination of T1 relaxation times via a flow‐sensitive alternating inversion recovery‐rapid acquisition with relaxation enhancement (RARE) sequence (TR = 3000 ms; TI between 6 and 6000 ms); (ii) assessment of T2 values using a multi‐slice multi‐echo (MSME) acquisition (TR = 4000 ms; TE from 4.8 to 61.2 ms); and (iii) T2* quantification through a multi‐gradient echo (MGE) sequence (TR = 4000 ms; TE spanning 2 to 17.47 ms). Longitudinal (r1), transverse (r2), and effective transverse (r2*) relaxivities were determined by linear regression of the relaxation rates (R1, R2, R2*) against the iron concentration.

### 4.5 Animals

Male Swiss and C57BL/6 mice (8 weeks old) were obtained from the Centre Universitaire de Ressources Biologiques (CURB) for this study. All procedures followed the ethical guidelines of the European Directive 2010/63/EU regarding animal welfare. The experimental protocol received formal validation from the Ministry of Higher Education and Research (approval #44410). The experiments have been conducted and reported following the ARRIVE (Animal Research: Reporting in Vivo Experiments) guidelines. Mice were housed under specific pathogen‐free conditions in a controlled environment maintained at 21°C and 55% humidity. The facility operated with a regulated pressure of 20–25 Pa, 100 lux brightness, and a 12‐h light/dark cycle, with food and water provided ad libitum. Before all procedures requiring anesthesia, mice were exposed to 5% isoflurane and subsequently maintained with 2% isoflurane delivered in a 70:30 ratio of nitrous oxide to oxygen. Mice received preparatory analgesia through a subcutaneous dose of buprenorphine (0.1 mg/kg), injected 30 min before surgery.

### 4.6 Intrastriatal LPS Injection

Inflammation was induced by an intrastriatal injection of lipopolysaccharide (1 µg per mouse; 0111:B4; Sigma–Aldrich). Anesthetized mice were placed in a stereotaxic frame, and an incision was made between the eyes to the nape of the neck. A small craniotomy was performed 2 mm lateral to the bregma, and the LPS (1 µL) was injected using a glass micropipette at a depth of 3 mm to reach the striatum.

### 4.7 In Vivo Immuno‐MRI

MPIOs were injected (1 mg/kg equivalent Fe) via a catheter through the tail vein 5 min before MRI acquisitions. MRI experiments were performed with a Pharmascan 7T/12cm system using surface coils (Bruker, Germany). To ensure immobility during MRI, animals were anesthetized with 5% isoflurane and maintained under anesthesia with 2% isoflurane. To visualize MPIOs, 3D FLASH gradient echo imaging was performed, yielding T2*‐weighted images with a spatial resolution of 78 µm × 78 µm × 150 µm (TE/TR = 8.6/50 ms, flip angle = 20°). Analyses were performed using ImageJ. The T2*‐weighted data are displayed as minimum intensity projections across three contiguous sections, providing a combined longitudinal resolution of 450 µm.

### 4.8 Immunohistochemistry

Animals were transcardially perfused with 15 mL of cold heparinized saline under deep anesthesia. Brains were harvested and kept for postfixation for 48 h in 2% paraformaldehyde (4°C), followed by 24 h of cryoprotection in 20% sucrose (4°C) before freezing in TissueTek. Brain sections (10 µm) were obtained via cryomicrotome, collected on poly‐lysine‐coated slides, and maintained at −80°C until use. Primary antibody incubation was performed for 2 h at room temperature with rat monoclonal anti‐mouse E‐selectin antibody (1:200; clone 96403 from R&D Systems), goat monoclonal anti‐mouse P‐selectin antibody (1:1000; clone 127933 from R&D Systems), rat anti‐mouse Ly6G antibody (1:200; clone 1A8 from BioLegend), Rat monoclonal anti‐CD3 antibody (1:200; Abcam ab11089) or rabbit polyclonal anti‐mouse Laminin antibody (1:200 from Abcam). Secondary detection was performed with Cy3‐, AF488‐, or AF647‐conjugated F(ab′)_2_ donkey anti‐rat, anti‐rabbit, or anti‐goat IgG. A Leica DM6000 epifluorescence system, coupled with a CoolSNAP camera and MM AF 2.2.0 software (Molecular Devices), was used to acquire 6 representative images per slide. All image processing and analysis were carried out using ImageJ.

### 4.9 Cranial Window Preparation

Cranial window was prepared using an established procedure [59]. To ensure aseptic conditions, all instruments were treated in a glass‐bead sterilizer (Elmasonic P 30 H, Singen, Germany). General anesthesia was induced in C57/Bl6 mice via an intraperitoneal (i.p.) administration of a medetomidine/fentanyl/midazolam cocktail (MMF; 0.5, 0.05, and 5 mg/kg, respectively). Once secured in a stereotaxic apparatus (RWD, Sugar Land, TX, USA), animals were placed on a feedback‐regulated heating pad (Harvard Apparatus, Holliston, MA, USA) to maintain physiological temperature. Vitamin A dulcis ointment (Allergan, Dublin, Ireland) was applied to the eyes to prevent drying during the procedure.

Following scalp disinfection with 70% ethanol, the skull was exposed by excising the skin with surgical scissors (FST, Heidelberg, Germany). Local analgesia was provided by topical application of 5% lidocaine (Aguettant, Langenfeld, Germany). After 1 min, an 11‐size scalpel (Surgeon, Gurgaon, India) was used to debride the periosteum. A 3‐mm biopsy punch (Integra LifeSciences, Princeton, NJ, USA) served to delineate the craniotomy site over the primary somatosensory cortex (coordinates relative to bregma: AP −0.9 mm, ML +3.0 mm). To prepare the bone surface, a light‐cured adhesive (iBond Self Etch, Hereaus Kulzer, Hanau, Germany) was applied and activated with an LED lamp (Foshan COXO Medical Instruments, Guangdong, China).

Craniotomy was performed by thinning the bone with a dental drill (BiosebLab, France) under continuous irrigation with sterile saline (Aguettant, Langenfeld, Germany). The resulting bone flap was gently lifted using angled forceps (S&T, FST, Heidelberg, Germany), followed by the delicate excision of the dura mater using SuperGrip forceps (FST, Heidelberg, Germany). To manage any cortical surface hemorrhage, a Spongostan gelatin sponge (Medical Devices, Soeborg, Denmark) was applied for 60 s. The window was then sealed by mounting a 3‐mm glass coverslip (VWR International, Radnor, PA, USA) with histoacrylic glue (Aesculap AG, Tuttlingen, Germany) and dental microbrushes (Microbrush International, Grafton, WI, USA). Structural reinforcement was provided by Tetric Evoflow A1 Fill dental cement (Ivoclar Vivadent, Schaan, Liechtenstein), and a custom‐made head‐post was integrated for future immobilization.

Post‐operative recovery was initiated by antagonizing the anesthesia with an i.p. injection of atipamezole (2.5 mg/kg), flumazenil (0.5 mg/kg), and naloxone (1.2 mg/kg). For analgesia, mice received subcutaneous buprenorphine (0.1 mg/kg; AnimalCare, York, UK) and were monitored in a recuperation chamber until fully awake.

### 4.10 Intravital Multiphoton Imaging

Intravital 2‐Photon images were scanned using Bruker Ultima 2P Plus (Billerica, Massachusetts, USA). Throughout the imaging session, mice were maintained under 1.5% isoflurane anesthesia and secured on a custom‐designed platform beneath the upright microscope objective. Fluorescence excitation was provided by a Chameleon Vision II laser (Coherent, Glasgow, Scotland) tuned to a wavelength of 920 nm, with an output power set to 300 a.u.

Images were captured through an XLUMPlanFL N 20×/1.0 W water‐immersion objective (Olympus, Tokyo, Japan). Signal detection was managed via GaAsP detectors equipped with RGB (Red, Green, Blue) filters, using a master gain of 500. Acquisition parameters were defined as follows: time‐lapse sequences (T‐series) were recorded using a Galvo scanner with a 4‐s frame interval and a 3.6 µs dwell time, while volumetric data (Z‐stacks) were acquired over a 50 µm depth. All images were recorded at a resolution of 1024 × 1024 pixels with an 8‐bit depth.

#### 4.10.1 Reconstruction

Three‐dimensional (3D) reconstruction of the obtained z‐stack was performed in Imaris 10.2 software (Oxford Instruments, Abingdon, UK). Using the artificial intelligence (AI)‐assisted “Surface” tool the vessels were reconstructed at “green” channel, while Nanoparticles were segmented using “Spots” tool in “blue” channel.

#### 4.10.2 Quantification

The number of particles was manually quantified using the Multi‐point tool in ImageJ [60] for each frame in a time‐lapse sequence acquired every 4 s from 0 to 160 s. For both LPS‐treated and naïve conditions, counts from five consecutive frames (20 s) were summed to obtain total particles per 20‐s interval (0–20 s, 20–40 s, …, up to 160 s). Vessel area was measured using the Freehand ROI tool, and particle counts were normalized to area and expressed as particles/mm^2^. A similar approach was applied to z‐series stacks (45 frames) for both conditions. Total particles were counted on the Maximum Intensity Projection of z‐stack, normalized by the measured vessel area, and expressed as particles/mm^2^.

### 4.11 Cell Culture

The bEnd.3 murine brain endothelial cell line (ATCC, Cellosaurus CVCL_0170) was used between passages 20 and 25. Cells were maintained in Dulbecco's Modified Eagle Medium (DMEM) containing 4.5 g/L glucose (Sigma–Aldrich), supplemented with 4 mm L‐glutamine (Gibco) and 10% non‐heat‐inactivated fetal calf serum (Biosera). Culture conditions were strictly controlled at 37°C within a humidified incubator set to a 10% CO_2_ atmosphere.

### 4.12 Flow‐Based Particles Adhesion Assay

bEnd.3 cells (50 000 cells/cm^2^) were grown in Ibidi µ‐Slide VI^0.4^ (Ibidi GmbH, Martinstreid, Germany) coated with collagen‐I (Sigma–Aldrich). Cells were cultured in static conditions in complete DMEM medium until reaching approximately 80% confluency. At this point, the culture medium was replaced with a 1:1 mixture of complete DMEM and serum‐free medium for endothelial cells (SFM, Gibco) to initiate serum deprivation. After 18 h of incubation, bEnd.3 cells were changed to SFM and exposed to murine TNFα (100 ng/mL; activated condition, #AF‐315‐01A, PeproTech) or PBS (unstimulated condition) for 18 h to induce endothelial E‐selectin expression. Control and activated endothelial cells were washed with fresh SFM media and connected to ibidi pump system (Ibidi) for the dynamic condition. A shear stress of 4 dyn cm^2^ was applied in unidirectional flow during 5 min after injection of either MPIO‐αE‐Selectin or MPIO‐IgG (25 µg/mL of iron concentration). Particles interactions with endothelial cells were monitored using Leica DMi8 microscope (Leica Microsystem) in brightfield mode. Time‐lapse imaging was recorded with a 20x objective, capturing images every 5 s over a duration of 5 min.

To generate the targeted wall shear stress (τ, expressed in dyn/cm^2^), the required flow rate (Φ, mL/min) was determined according to the manufacturer's specifications for the Ibidi µ‐slide VI 0.4. The calculation followed the equation τ = η × 176.1 × Φ, where η represents the dynamic viscosity of the serum‐free medium (0.010dyn·s/cm 2 at 22°C). This formula integrates both the fluid properties and the specific internal geometry of the flow chamber.

#### 4.12.1 Immunofluorescence

Endothelial cells were fixed, immediately after flow, with 4% paraformaldehyde (PFA) for 20 min and washed three times with PBS. Cells were then permeabilized for 5 min with 0.1% Triton X‐100‐PBS solution and incubated for 20 min in 1% BSA‐PBS blocking buffer at room temperature. To visualize bound MPIO particles (either αE‐Selectin or IgG), Alexa Fluor 647‐conjugated Donkey Anti‐Rat IgG (2 µg/mL, Jackson ImmunoResearch Laboratories) was incubated during 1 h in 1% BSA‐PBS blocking buffer. To visualize the actin cytoskeleton, cells were stained with iFluor488‐phalloidin (Abcam), a bicyclic heptapeptide with high affinity for F‐actin filaments. The flow channels were subsequently filled with ibidi mounting medium containing DAPI (Ibidi) for nuclear staining. Confocal microscopy was performed using a Leica SP5 system equipped with a 40x objective, capturing images at a 1024 × 1024 pixels resolution with a 0.40 µm Z‐step. Image processing, analysis, and reconstructions were conducted using ImageJ 1.5 software (National Institutes of Health).

### 4.13 Magnetic Particle Imaging

Mice were anesthetized and received an intravenous injection of MPIO‐αE‐selectin 24 h after intrastriatal LPS administration. Magnetic particle imaging (MPI; Momentum CT, Magnetic Insight, Inc., Alameda, CA, USA) was performed 24 h after MPIO injection using a magnetic gradient strength of 5.7 T/m. Multichannel mode was selected, and 3D MPI datasets were acquired with 21 projections and a field of view (FOV) of 10 cm × 6 cm × 6 cm (slice thickness: 0.25 mm). To perform ex vivo imaging, target organs were harvested immediately following the in vivo MPI session. The organs were collected without prior perfusion, weighed individually, and arranged on Parafilm. Subsequently, two‐dimensional MPI scans were acquired for each specific organ.

### 4.14 ELISA

Brain hemispheres were lysed with RIPA buffer (Pierce RIPA Buffer, 89900) containing protease inhibitor cocktail (P8340, Sigma–Aldrich) and phosphatase inhibitor cocktail (P5726, Sigma–Aldrich). Lysates were then centrifuged for 15 min at 12 000 g at 4°C. Protein concentrations were measured using BCA protein assay kit (Pierce BCA Protein Assay Kit, 23225). The level of E‐selectin in the brain hemisphere was analyzed by enzyme‐linked immunosorbent assay (ELISA; Duoset DY575; R&D Systems) according to the manufacturer's instructions.

### 4.15 Permanent Middle Cerebral Artery Occlusion

The permanent middle cerebral artery occlusion (MCAO) was performed as described before [61]. Following induction of anesthesia, mice were head‐fixed in a stereotaxic apparatus. A focal craniotomy was then carried out to provide surgical access to the MCA. The MCA was occluded using a piece of Whatman filter paper strip soaked in freshly prepared aluminum chloride, placed on the endothelium for 4 min. The regional cerebral blood velocity was monitored by laser Doppler flowmetry (Oxford Optronix). The MCA was considered occluded when cerebral blood flow decreased to below 20% of baseline.

### 4.16 Flow Cytometry

#### 4.16.1 In Vitro E‐Selectin Expression

bEnd.3 cells were seeded at a density of 5 × 10^4^ cells/mL and stimulated with 100 ng/mL of TNF‐α for 18 h in serum‐free medium. After stimulation, cells were detached using Accutase and resuspended at a concentration of 1 × 10^6^ cells/mL in PBS‐BSA 1% buffer. Fc receptor blocking was performed by incubating the cells with an anti‐CD16/32 antibody for 10 min at 4°C. Cells were then stained with an E‐selectin primary antibody (25 µg/mL, BioCell) for 30 min at 4°C. An isotype control using a rat IgG2b antibody at the same concentration was included. After washing, cells were incubated with a donkey anti‐rat Alexa Fluor 488‐conjugated secondary antibody (4 µg/mL, Jackson ImmunoResearch) for 45 min at room temperature. Following the two washing steps, the viability dye 7‐AAD was added to exclude dead cells. Samples were acquired on a FACSVerse flow cytometer (BD Biosciences) using FACSuite software. A total of 50 000 events were recorded after gating based on forward scatter, side scatter, and live cells. Alexa Fluor 488 fluorescence was detected in the FITC channel, and data were analyzed using representative dot plots showing the percentage of E‐selectin‐positive cells.

#### 4.16.2 Characterization of Brain Immune Cell Infiltration

After perfusing the mice transcardially with ice‐cold PBS, the brains were dissected, finely chopped, and homogenized in Hanks’ balanced salt solution containing 15 mm HEPES and 0.54% glucose using a Potter tissue grinder. The resulting homogenates were then layered onto a 37% Percoll gradient and centrifuged at 800 g for 30 min at 4°C with no brake applied. CNS leukocytes, forming a pellet at the bottom of the tube, were carefully collected, rinsed in PBS with 2% fetal calf serum, and prepared for staining. To prevent non‐specific binding, Fc receptors were blocked using CD16/CD32 (BD Biosciences, 553142) for 10 min at 4°C prior to antibody staining. The cells were then labeled with a panel of antibodies targeting CD45 (APC‐Cy7, BD Biosciences), Thy1.2 (BV421, BD Biosciences), Ly6G (FITC, BD Biosciences), CD4 (BV510, BD Biosciences), CD8a (Pe‐Cy7, BD Biosciences), CD206 (APC, BioLegend), and P2Y12R (PE, BioLegend). Incubation was performed at 4°C for 20 min, except for CD206, which required a 45‐min staining period. Following the washing step, samples were analyzed on a FACSVerse flow cytometer using BD FACSuite 1.0.6 software. The resulting data were processed with FlowJo 7.6.5 software (TreeStar).

### 4.17 Statistical Analysis

Statistical significance was defined as *p* < 0.05. The choice between parametric and non‐parametric methods was determined by the distribution of the data. For two‐group comparisons, we employed the Student's *t*‐test for normally distributed datasets and the Mann–Whitney U test for non‐normal distributions. In experiments involving more than two groups, a one‐way ANOVA followed by Holm–Šídák's or Tukey's post‐hoc tests was performed for normally distributed data, whereas the Kruskal–Wallis test with Dunn's multiple comparison test was used for non‐normal data. Finally, the association between variables was assessed using either Pearson's or Spearman's correlation coefficients, depending on the data distribution.

## Funding

This work was performed in a facility of the France Life Imaging network (grant ANR‐11‐INBS‐0006). We acknowledge the France‐BioImaging infrastructure supported by the French National Research Agency (ANR‐24‐INBS‐0005 FBI BIOGEN). APF was funded by the WINNINGNormandy Program, supported by the Normandy Region and the European Union's Horizon 2020 research and innovation programme under the Marie Skłodowska‐Curie grant agreement No. 101034329. MG is an Impulscience laureate of the Bettencourt Schueller Foundation. This work was supported by the French government through the National Research Agency (ANR) under the France 2030 program, as part of the CaeSAR project (reference ANR‐23‐EXES‐0001), the FlaMRIng project (reference ANR‐20‐CE19‐0032‐01), and by the Normandy Region (FEDER‐FSE 2021–2027) through the MAGENTA project (reference 014C399BP). We gratefully acknowledge support from the JPND Research Program through the REBALANCE project (grant number ANR‐22‐JPWG‐0004) and the ERANET‐Neuron JTC2022 program through the IMatrix project. Figures were created using BioRender.com.

## Conflicts of Interest

The authors declare no conflicts of interest.

## Supporting information




**Supporting File 1**: adhm71325‐sup‐0001‐SuppMat.pdf

**Supporting File 2**: Movie_S1_bEnd3_TNF_MPIO@Esel

**Supporting File 3**: Movie_S2_bEnd3_TNF_MPIO@IgG

**Supporting File 4**: Movie_S3_bEnd3_Unstim_MPIO@Esel

**Supporting File 5**: Movie_S4_bEnd3_Unstim_MPIO@IgG

## Data Availability

The data that support the findings of this study are available from the corresponding author upon reasonable request.
