# Supplementary material for: Neutrophil‐Mimetic MRI Enables Ultra‐Early Detection of Vascular Inflammation After Stroke
Source: Adv Healthc Mater. 2026 Jun 8;15(25):e71325. doi: 10.1002/adhm.71325 (PMC13331590; doi:10.1002/adhm.71325)
Supplement: Supplementary file 1 — Supporting File 1: adhm71325‐sup‐0001‐SuppMat.pdf [file ADHM-15-0-s002.pdf]

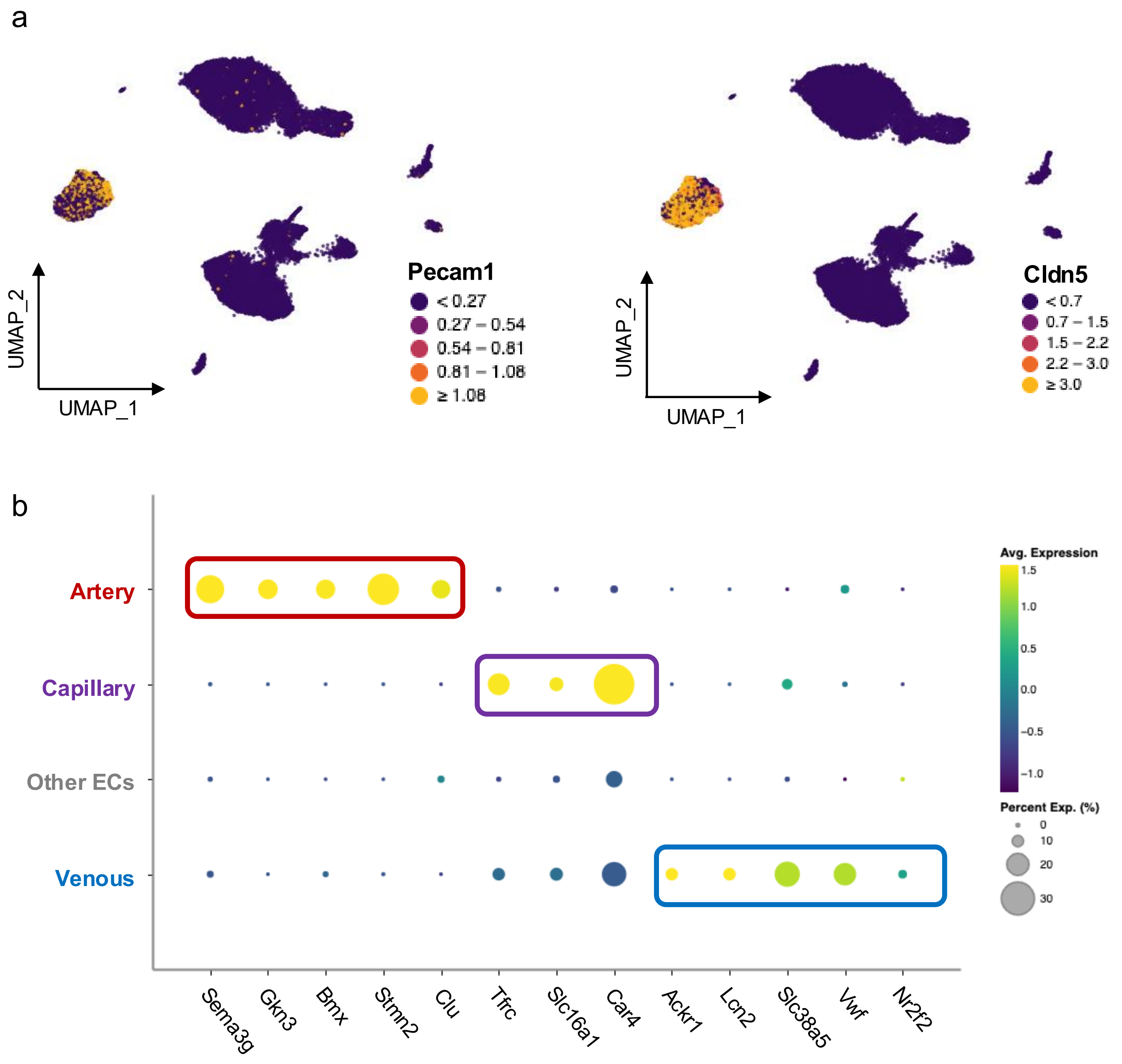

Supplementary Figure S1

Supplementary Figure S1: Transcriptomic profiling identifies endothelial cell subtypes. (a) Expression of known endothelial markers (*Pecam1* and *Cldn5*) projected onto UMAP. **(b)** Dot plot of known arteriovenous zonation markers across endothelial cell populations.

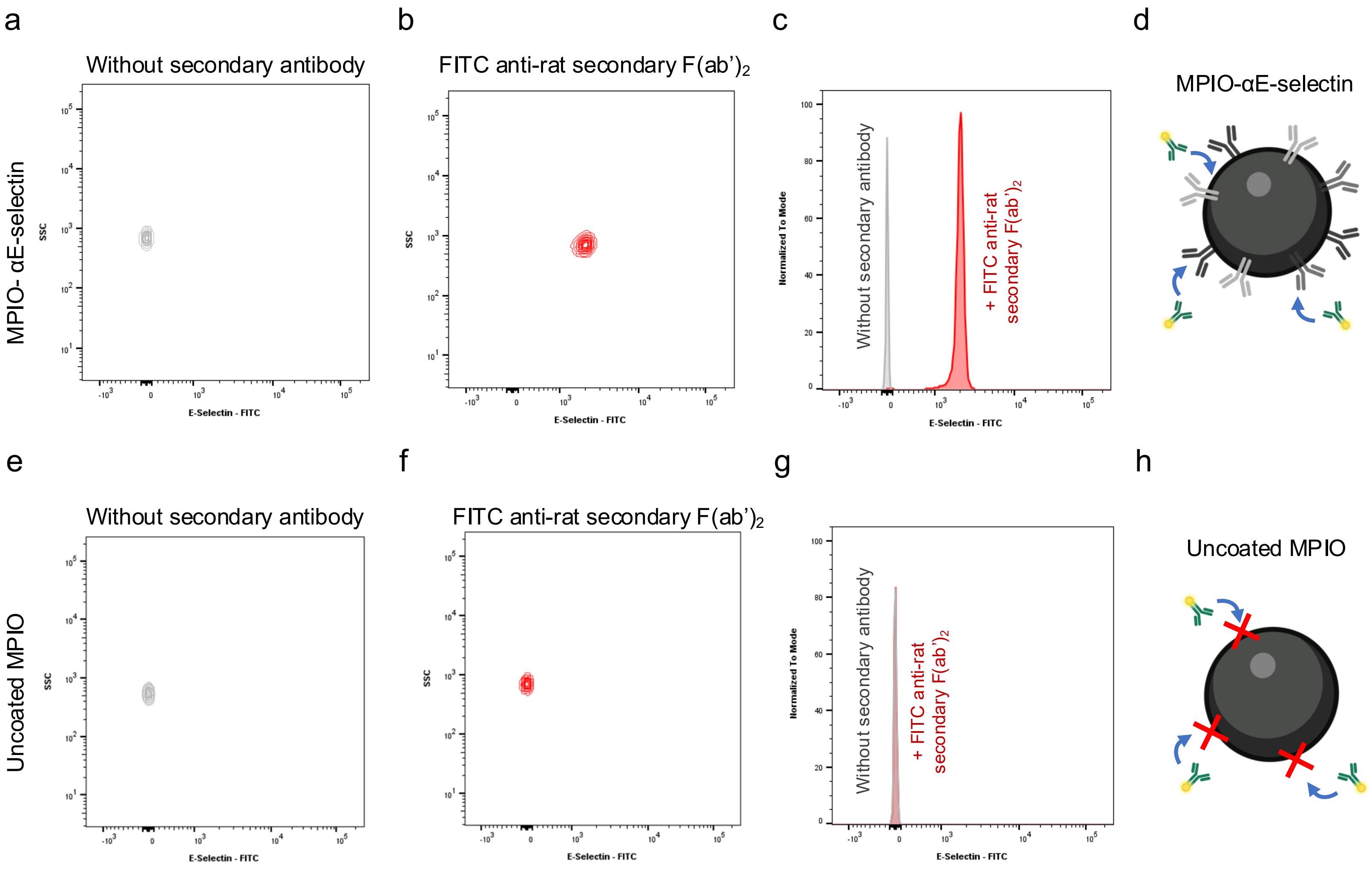

Supplementary Figure S2

Supplementary Figure S2: Effective coating of MPIOs with anti-E-selectin monoclonal antibodies assessed by flow cytometry. 2D density plots of MPIO-αE-selectin **(a)** without and **(b)** with FITC anti-rat secondary $F(ab')_2$ antibodies. **(c)** Histogram analysis of the data shown in panels a and b, demonstrating a fluorescence shift in the MPIO-αE-selectin population when incubated with the secondary $F(ab')_2$ antibodies, consistent with secondary antibody binding to MPIO-αE-selectin. **(d)** Schematic illustration summarizing panels a-c. **(e-h)** Same analyses performed using uncoated MPIOs. As expected, FITC anti-rat $F(ab')_2$ antibodies did not bind uncoated MPIOs, resulting in no fluorescence shift upon incubation with the secondary antibodies.

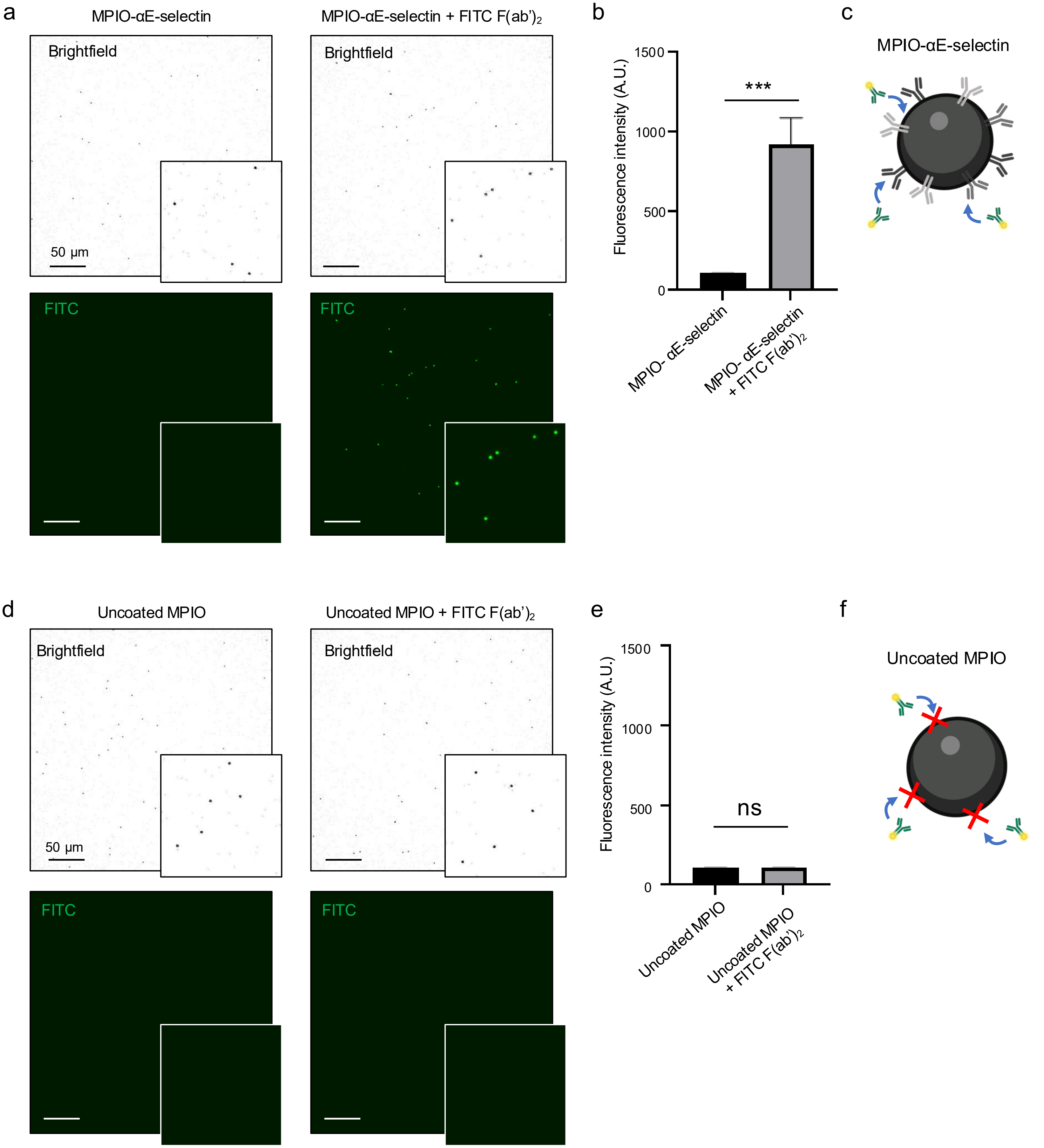

Supplementary Figure S3

Supplementary Figure S3: Effective coating of MPIOs with anti-E-selectin monoclonal antibodies assessed by fluorescence microscopy. (a) Representative microscopy images of MPIO-αE-selectin (top: brightfield reflectance; bottom: FITC fluorescence, green) incubated without (left) or with (right) FITC anti-rat secondary $F(ab')_2$ antibodies. **(b)** Quantification of fluorescence intensity (n = 24-28 per group), showing significantly higher fluorescence in the presence of the FITC anti-rat $F(ab')_2$, consistent with binding of the secondary antibodies to MPIO-αE-selectin. Data are presented as mean ± SD. Statistical analyses were performed using Mann-Whitney U-test. *** $P < 0.001$. **(c)** Schematic illustration summarizing panels a and b. **(d-f)** Same analyses performed on uncoated MPIOs. The FITC anti-rat secondary $F(ab')_2$ antibodies did not bind to uncoated MPIOs, resulting in similar fluorescence levels whether or not the secondary antibody was added, as shown in panel e (n = 38-46 per group).

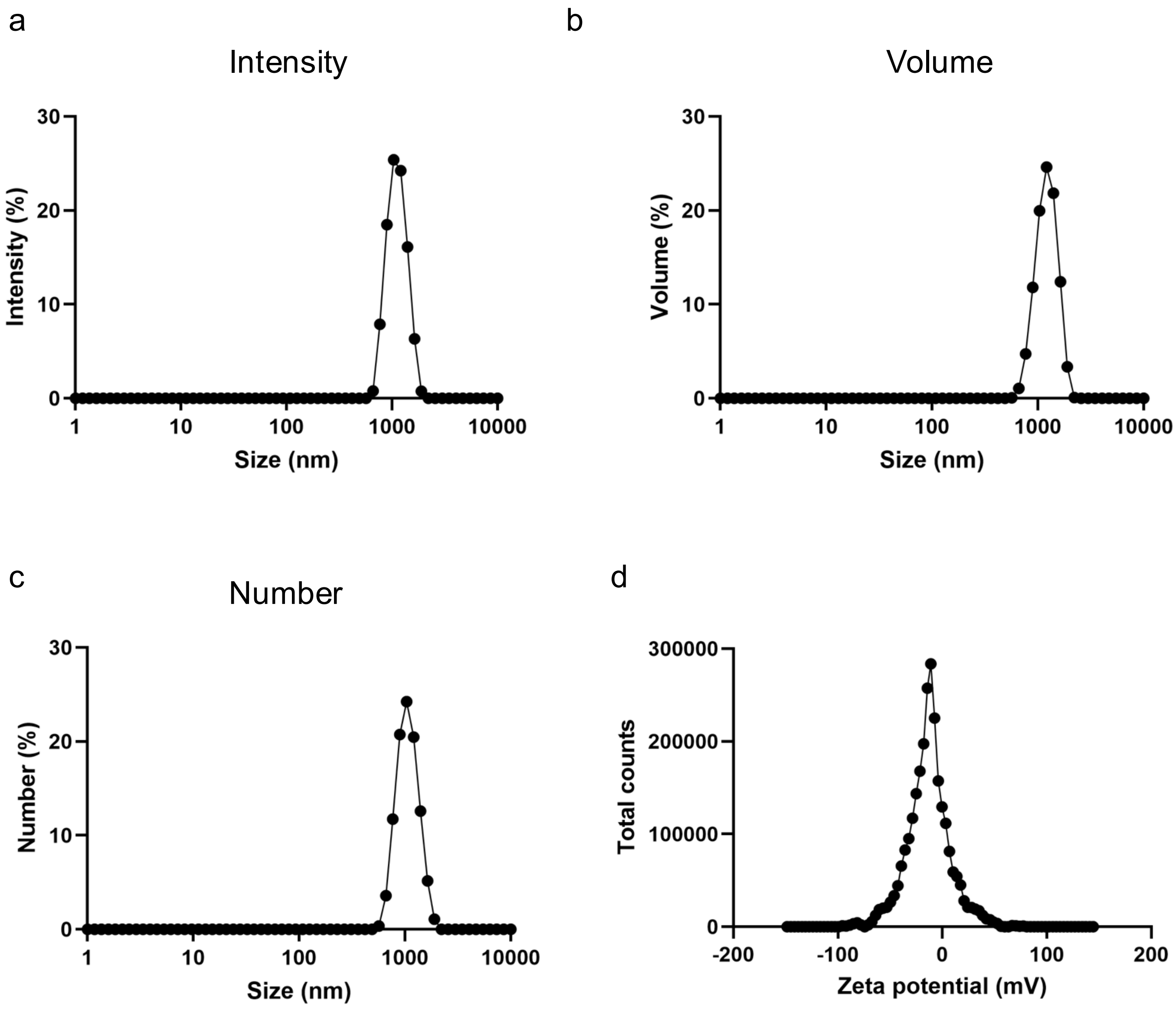

Supplementary Figure S4

Supplementary Figure S4: Dynamic light scattering measurements of MPIO-αE-selectin. (a) Intensity-weighted size distribution histogram. **(b)** Volume-weighted size distribution histogram. **(c)** Number-weighted size distribution histogram. **(d)** Zeta-potential histogram. The mean hydrodynamic diameter was 1090 nm, with a polydispersity index of 0.058 and a zeta potential of -12.7 mV.

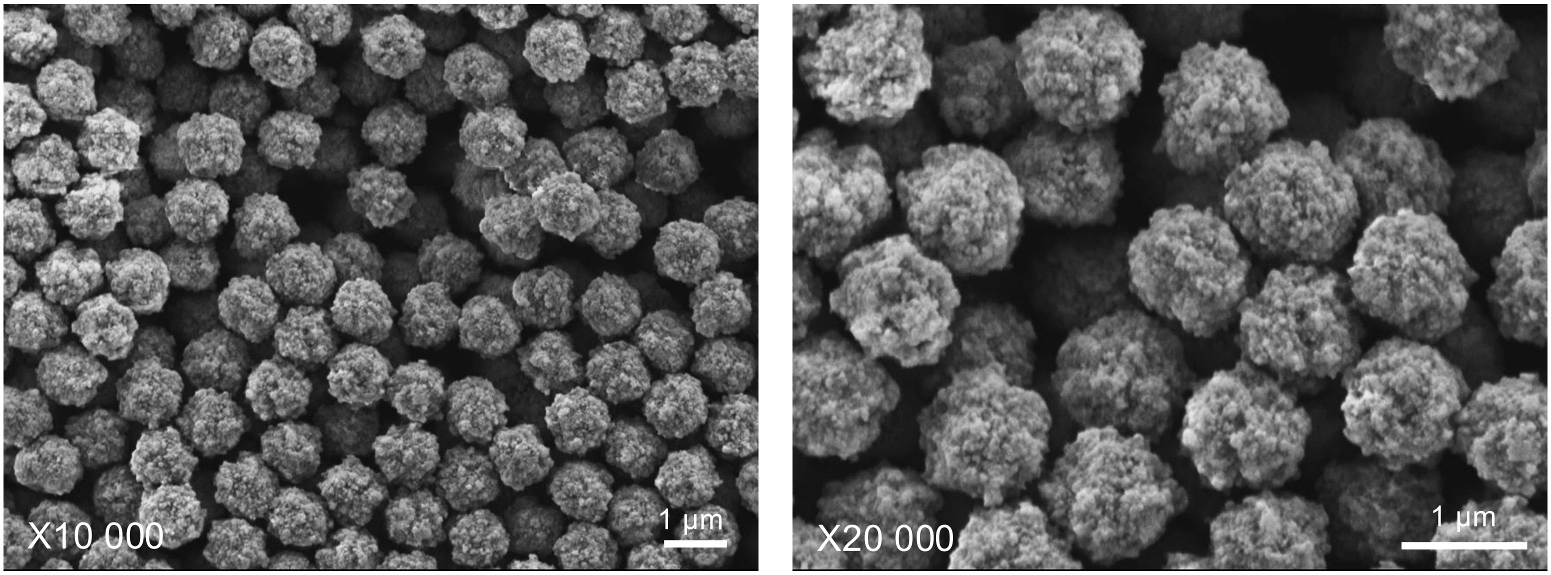

Supplementary Figure S5

Supplementary Figure S5. Representative high-resolution scanning electron microscopy (SEM) image of microparticles of iron oxide (MPIO). The SEM visualization confirms the spherical morphology and micrometer-scale dimensions of the particles, as well as their overall uniform size distribution in suspension.

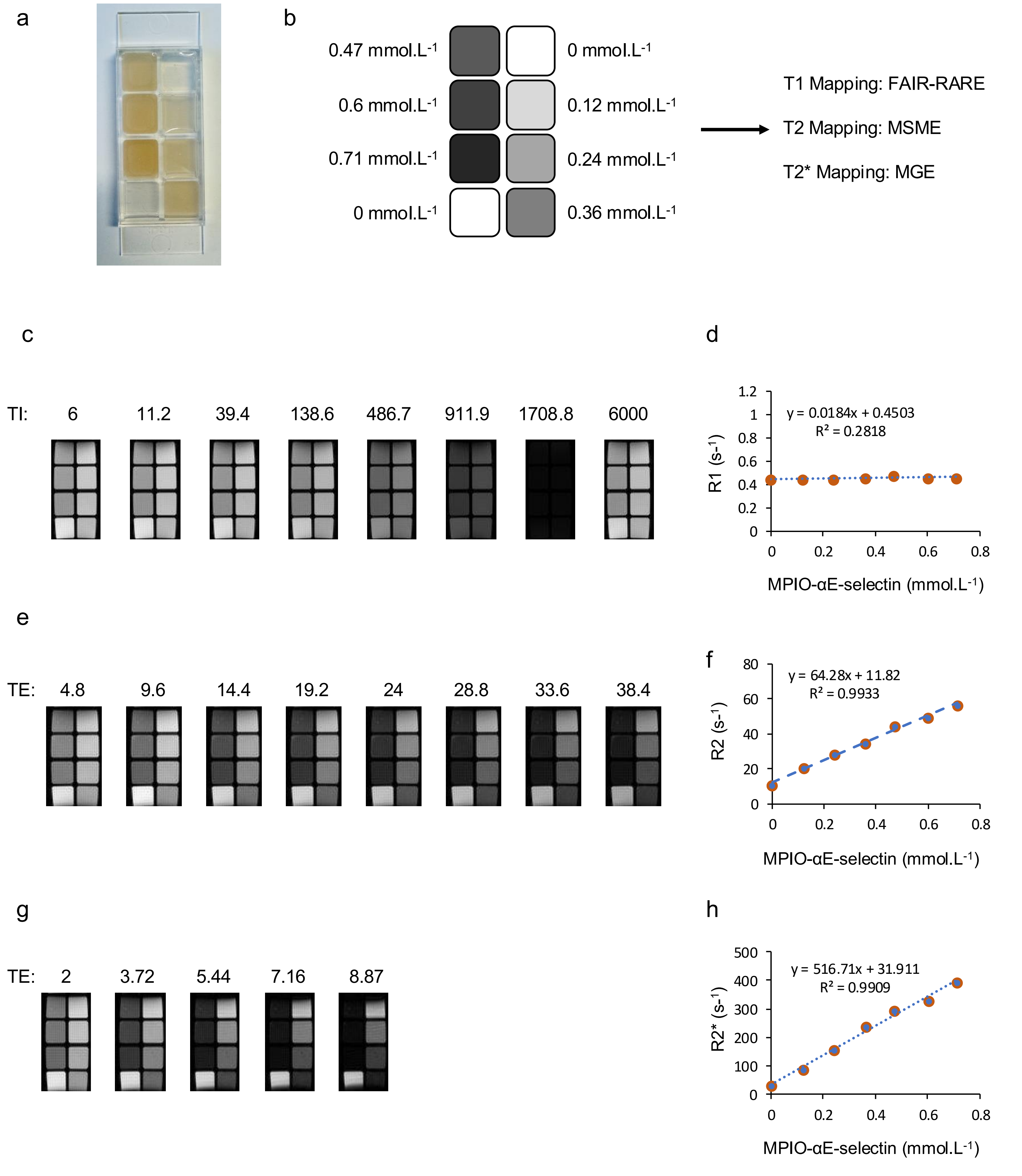

Supplementary Figure S6

Supplementary Figure S6. Relaxivity measurements of micron-sized particles of iron oxide (MPIO) performed using a 7 T preclinical MRI system. (a) Photograph of the multi-well plate containing MPIO embedded in 2% agarose gels at increasing iron concentrations. **(b)** Schematic overview of the experimental setup used for relaxivity measurements. **(c)** Representative FAIR-RARE images acquired at different inversion times (TI). **(d)** Corresponding calculation of the longitudinal relaxivity (R1) of MPIO. **(e)** Representative multi-slice multi-echo (MSME) images acquired at different echo times (TE). **(f)** Corresponding calculation of the transverse relaxivity (R2) of MPIO. **(g)** Representative multi-gradient echo (MGE) images acquired at different echo times (TE). **(h)** Corresponding calculation of the effective transverse relaxivity (R2*) of MPIO. Note that panels (c), (e), and (g) display the same multi-well plate imaged at different time points of the MRI pulse sequences to illustrate the signal decay/recovery used for relaxivity calculations.

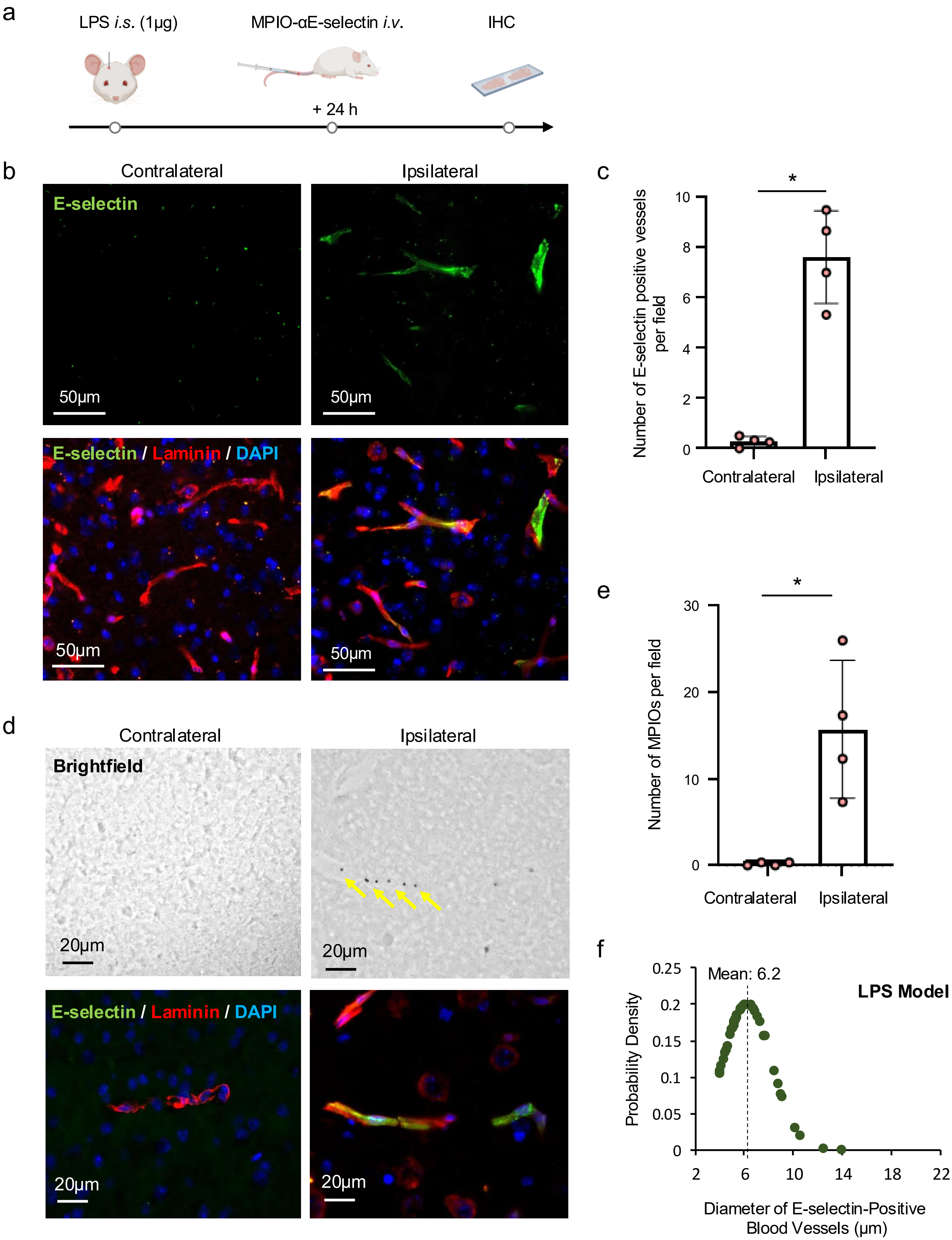

Supplementary Figure S7

Supplementary Figure S7: Characterization of E-selectin expression and MPIO-αE-selectin binding. (a) Schematic representation of the experimental procedure. *i.s.*, intrastriatal; *i.v.*, intravenous. Illustration created using BioRender.com. **(b)** Representative immunohistochemical images of E-selectin expression in brain blood vessels of mice injected with 1 µg of LPS in the right striatum. **(c)** Quantification of the number of E-selectin-positive vessels per field. (mean ± SD, n = 12-15 fields from 4 different mice per group). Statistical analyses were performed using Mann-Whitney U-test. * $P < 0.05$. **(d)** Representative immunohistological images of E-selectin expression and MPIO-αE-selectin in brain blood vessel of mice injected with 1 µg of LPS in the right striatum. **(e)** Quantification of the number of MPIO-αE-selectin per field (mean ± SD, n = 12 fields from 4 different mice per group). Statistical analyses were performed using Mann-Whitney U-test. * $P < 0.05$. **(f)** Probability density curve of the sizes of E-selectin-positive vessels.

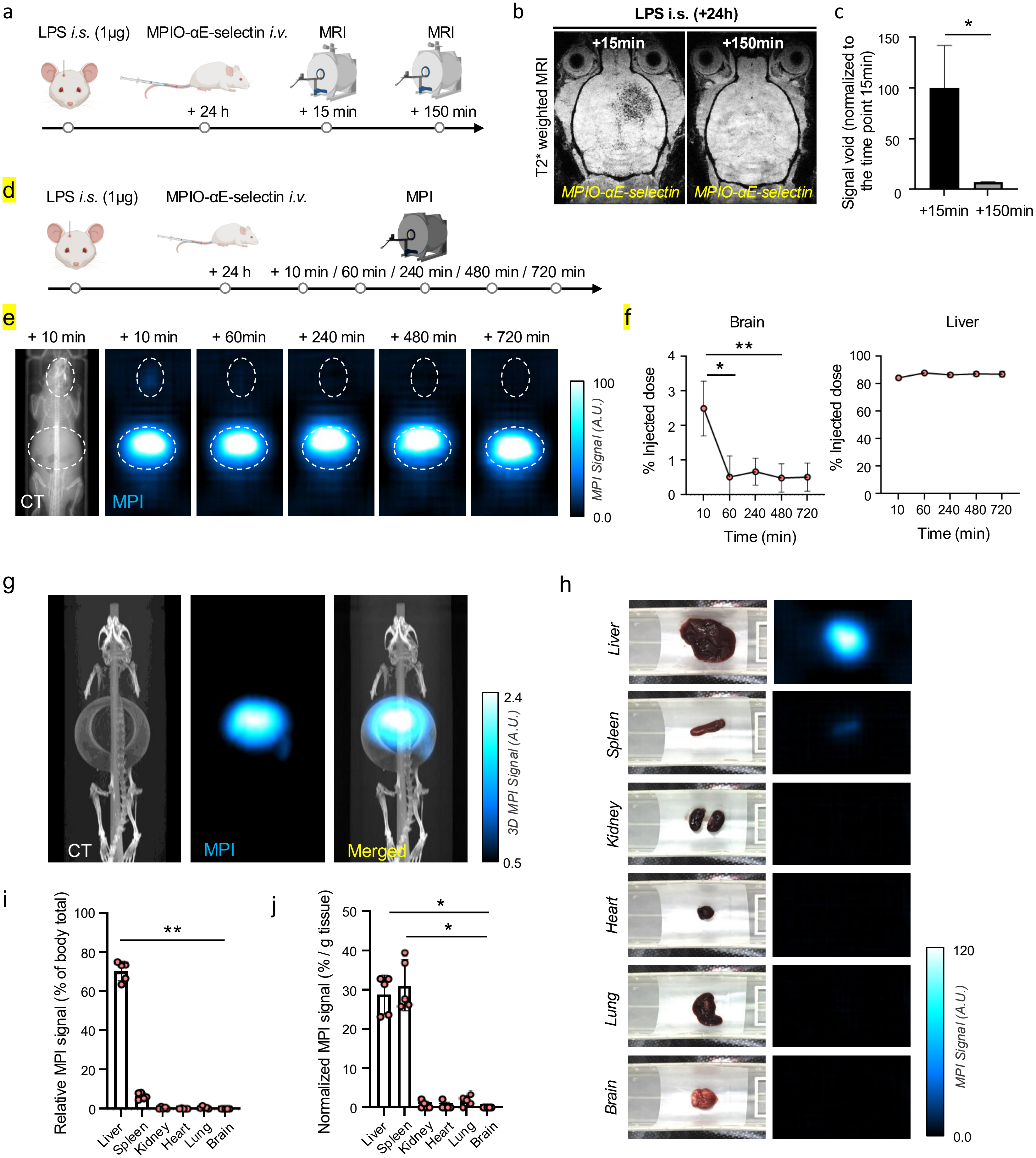

Supplementary Figure S8

Supplementary Figure S8: MPIO-αE-selectin exhibits a short binding duration. (a) Schematic representation of the experimental procedure. *i.s.*, intrastriatal; *i.v.*, intravenous. Illustration created using BioRender.com. **(b)** Representative T2*-weighted images obtained 15min and 150min after intravenous administration of MPIO targeted against E-selectin in mice treated with LPS. **(c)** Corresponding quantification of MPIO-αE-selectin-induced signal void (mean ± SEM, n = 4). Statistical analyses were performed using Mann-Whitney U-test. * $P < 0.05$. **(d)** Experimental design. i.s., intrastriatal; i.v., intravenous; MPI, magnetic particle imaging. Illustration created with BioRender.com. **(e)** Representative 2D MPI-CT images at different time points after intravenous injection of MPIO-αE-selectin. **(f)** Corresponding quantification of the 2D MPI signal (mean ± SD; n=5 per time point, paired data). Statistical analyses were performed using the Friedman test followed by Dunn's multiple comparison test. *$P < 0.05$, **$P < 0.01$. **(g)** 3D MPI images acquired 24 h after intravenous administration of E-selectin-targeted MPIOs in LPS-treated mice. **(h)** *Ex vivo* MPI images of isolated organs collected 24 h post-injection. **(i)** Quantification of total MPI signal per organ, expressed as a percentage of the total body signal (mean ± SD, n = 5). Friedman test followed by Dunn's multiple comparisons test. * $P < 0.05$. **(j)** Quantification of MPI signal normalized to tissue weight (% per gram of tissue; mean ± SD, n = 5). Friedman test followed by Dunn's multiple comparisons test. * $P < 0.05$.

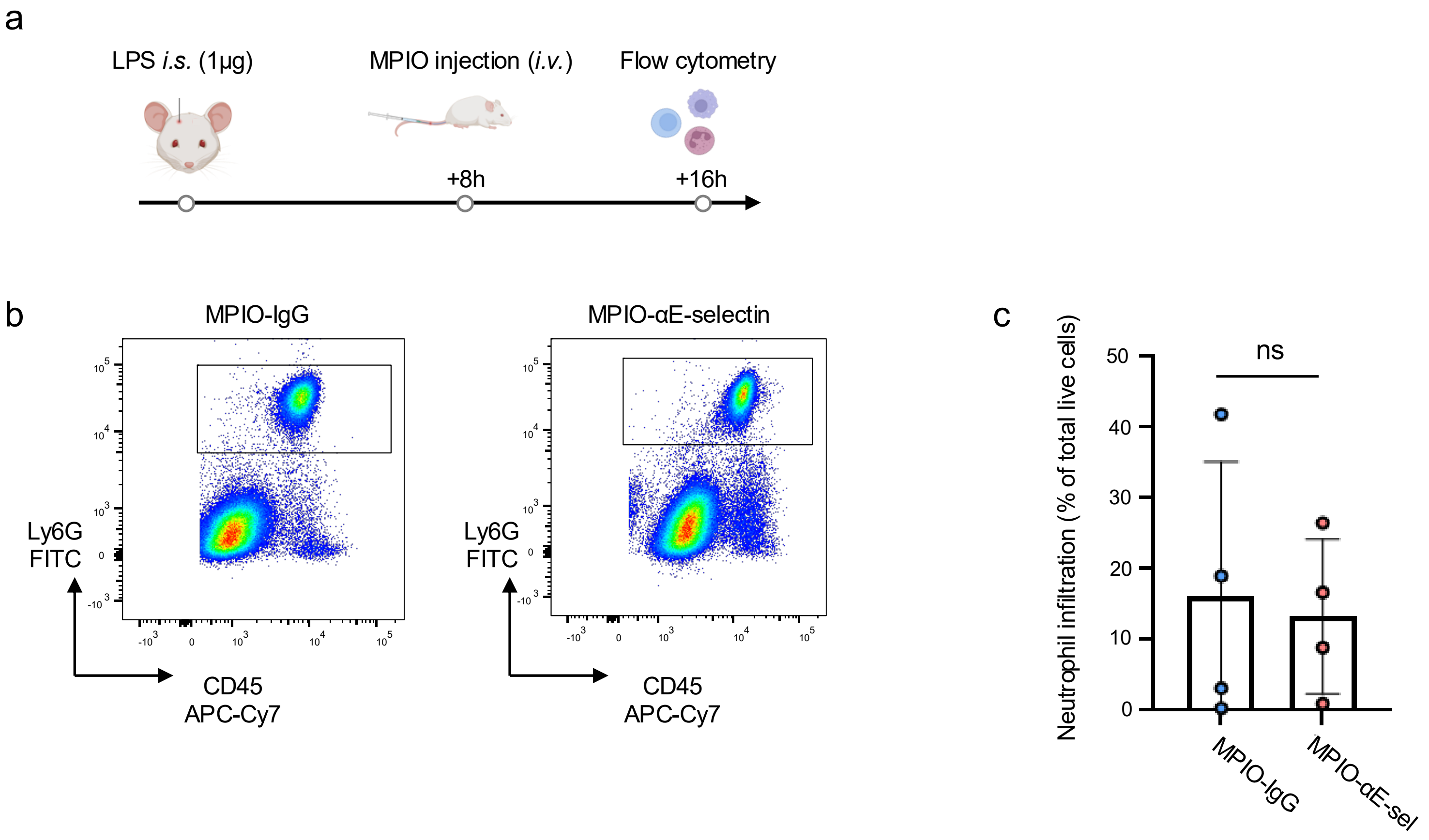

Supplementary Figure S9

Supplementary Figure S9: Impact of MPIO-αE-selectin injection on neutrophil infiltration. (a) Schematic representation of the experimental procedure. *i.s.*, intrastriatal; *i.v.*, intravenous. Illustration created using BioRender.com. **(b)** Representative flow cytometry dot plots showing neutrophil frequency in the brain of LPS-treated mice injected with MPIO-IgG or MPIO-αE-selectin. **(c)** Quantification of neutrophil infiltration in the brain of LPS-injected mice treated with MPIO-IgG or MPIO-αE-selectin (n = 4 per group). Data are expressed as a percentage of total live cells and presented as mean ± SD. Statistical analysis was performed using the Mann-Whitney U-test.

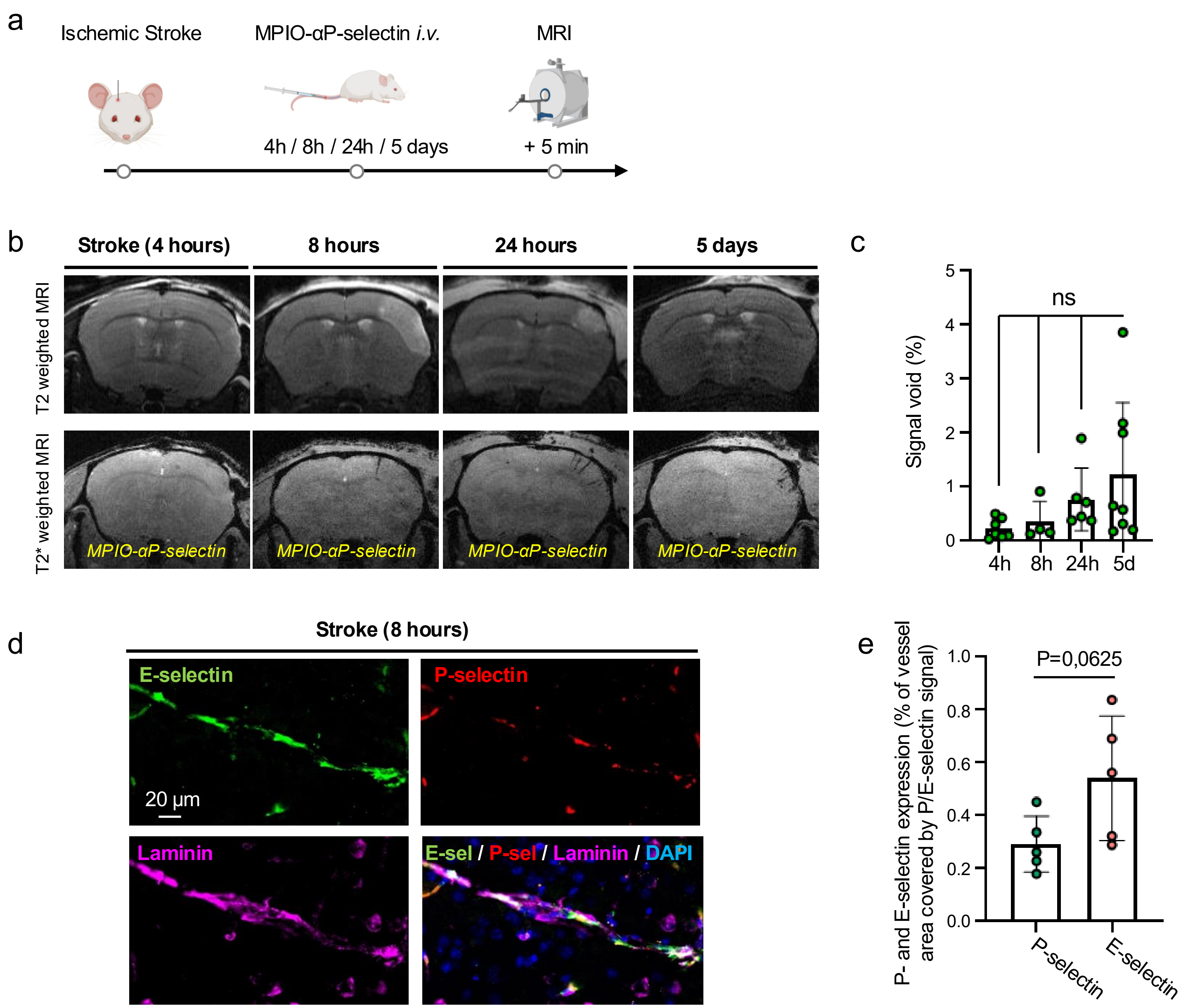

Supplementary Figure S10

Supplementary Figure S10: Immuno-MRI targeting P-selectin in an ischemic stroke model. (a) Schematic representation of the experimental procedure. *i.v.*, intravenous. Illustration created using BioRender.com. **(b)** Representative T2*-weighted images obtained after intravenous administration of MPIO-αP-selectin performed 4, 8, 24 hours, and 5 days post-MCAO. **(c)** Quantification of MPIO-αP-selectin-induced signal void in the right cortex (mean ± SD, *n* = 4-8). Statistical analysis was performed using the Kruskal-Wallis Test + Dunn's multiple comparison test. **(d)** Representative immunohistochemical images of P- and E-selectin expression in brain blood vessels 8 hours post-MCAO. **(e)** Quantification of vascular P- and E-selectin expression in the ischemic hemisphere at 8 h after MCAO. Data show the percentage of vessel area covered by P/E-selectin signal (mean ± SD, *n* = *5* mice per group). Statistical analysis was performed using the Wilcoxon test.

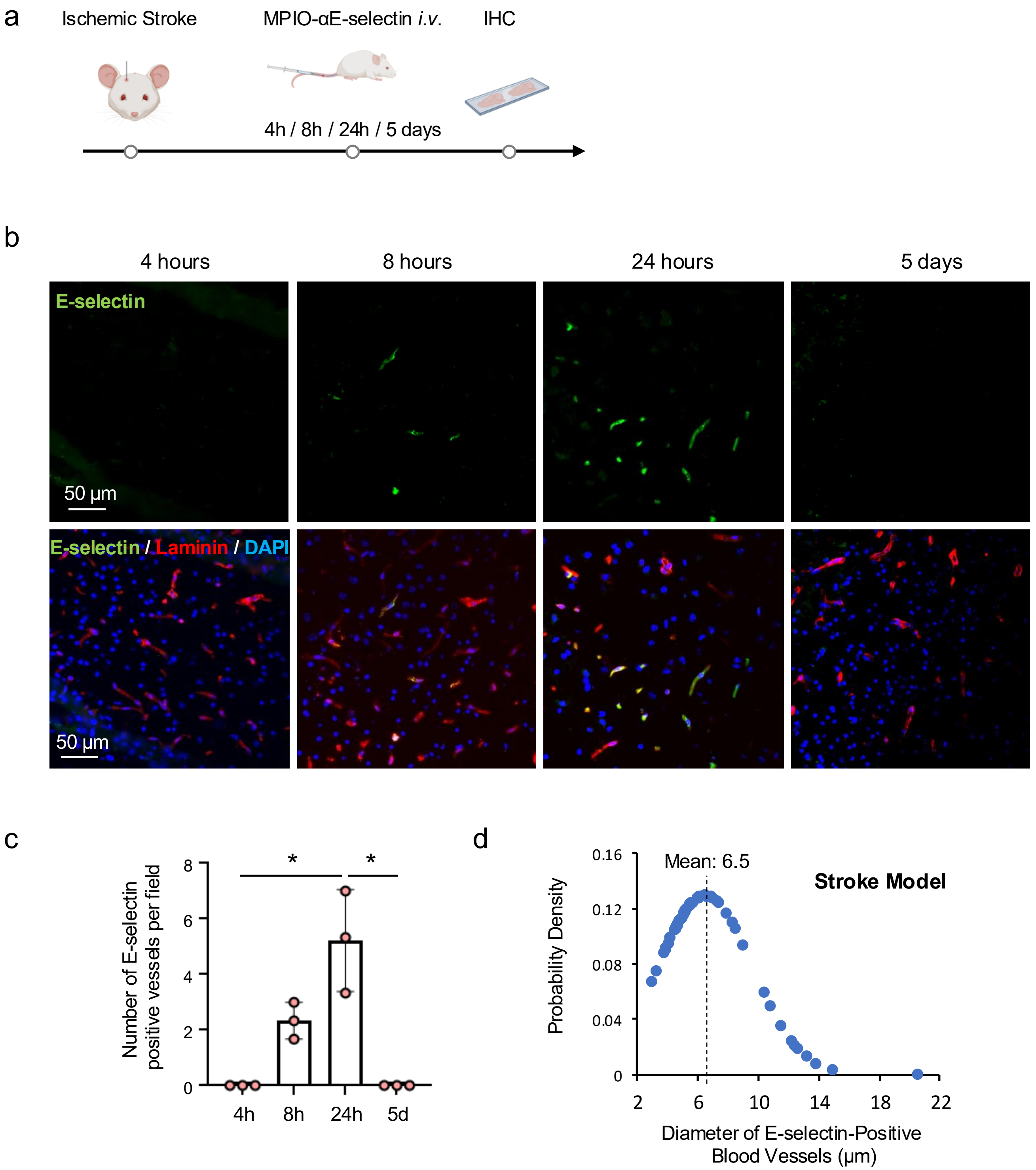

Supplementary Figure S11

Supplementary Figure S11: Characterization of E-selectin expression in a model of ischemic stroke. (a) Schematic representation of the experimental procedure. *i.v.*, intravenous. Illustration created using BioRender.com. **(b)** Representative immunohistochemical images of E-selectin expression in brain blood vessels at 4, 8, 24 hours, and 5 days post-MCAO. **(c)** Quantification of the number of E-selectin-positive vessels per field (mean ± SD, *n* = 3 mice per group). Statistical analysis was performed using the Kruskal-Wallis Test + Dunn's multiple comparison test. **(d)** Probability density curve of E-selectin-positive vessels sizes.

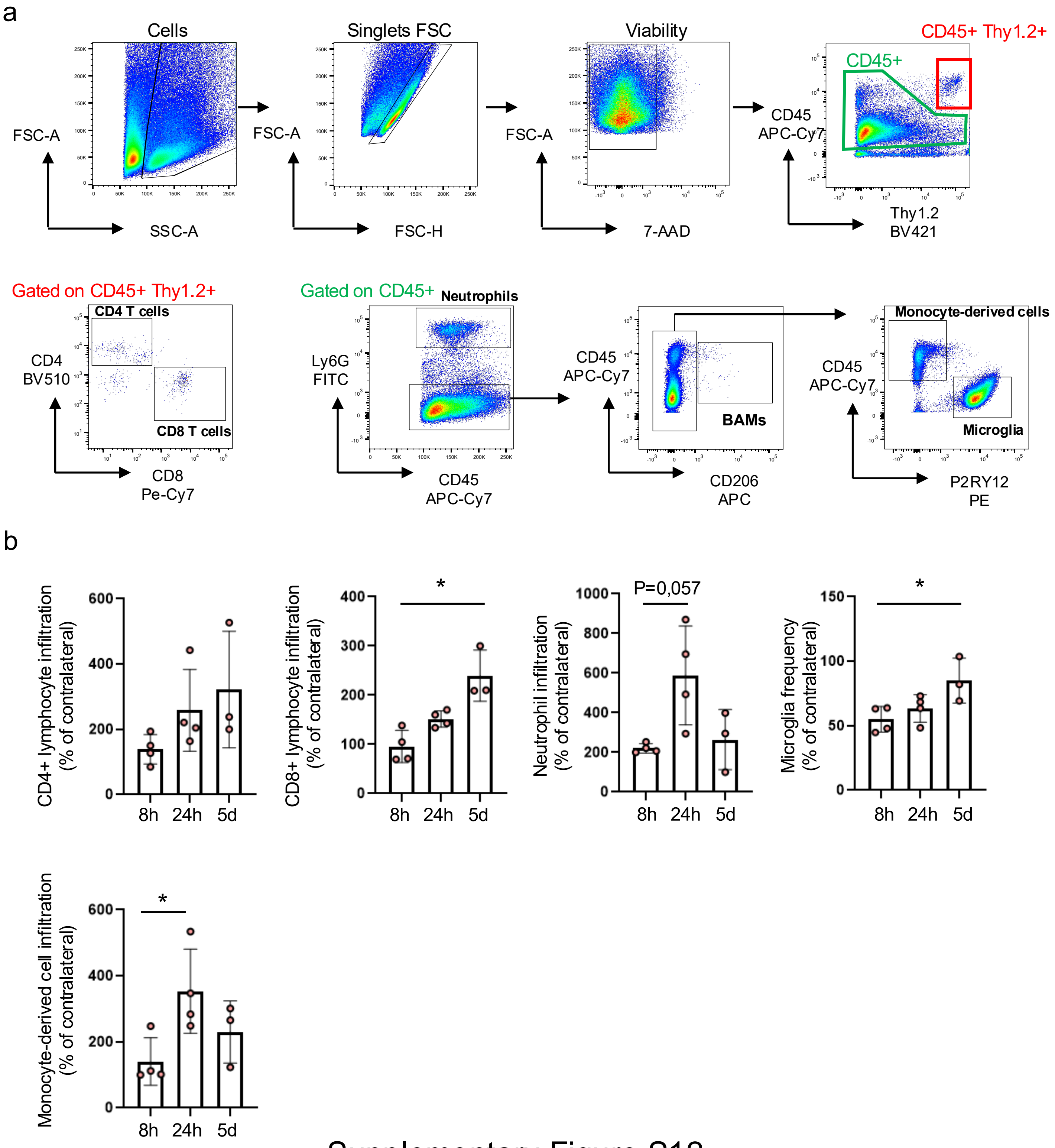

Supplementary Figure S12

Supplementary Figure S12: Flow cytometry gating strategies for quantifying immune cell infiltration in an ischemic stroke model. (a) Representative flow cytometry dot plots and gating strategy used to quantify CD4+ and CD8+ lymphocytes, neutrophils, microglia and monocyte-derived cells in post-MCAO brains. BAMs: Border-associated macrophages. **(b)** Flow cytometry quantification of CD4+ and CD8+ lymphocytes, neutrophils, microglia, and monocyte-derived cells in post-MCAO brains (mean ± SD, *n* = 3-4). Statistical analysis was performed using the Kruskal-Wallis test + Dunn's multiple comparison test.

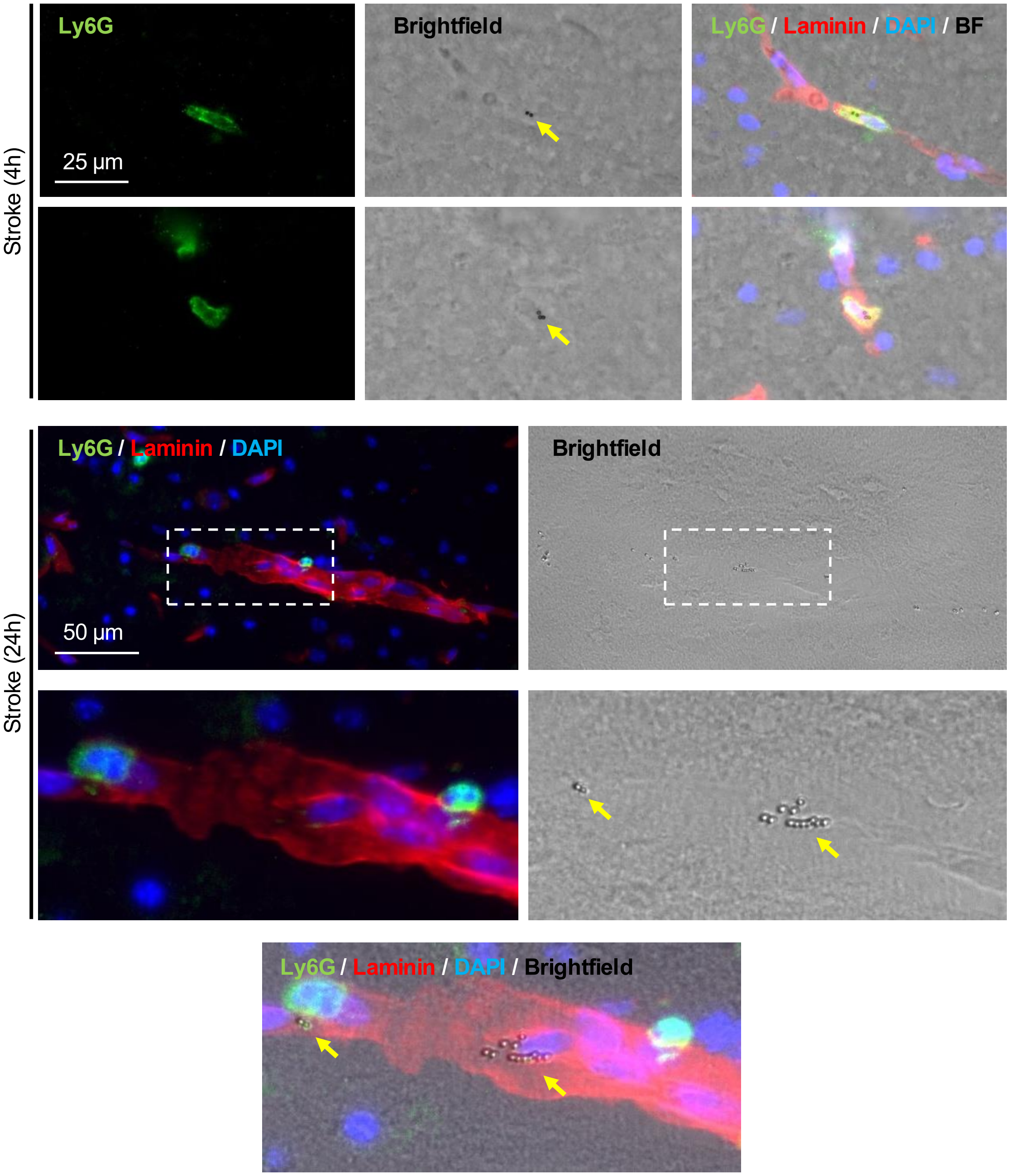

Supplementary Figure S13

Supplementary Figure S13: MPIO-αE-selectin binds to activated endothelium at neutrophil adhesion sites. Representative immunofluorescence images of MPIO-αE-selectin and Ly6G+ neutrophils in brain blood vessels at 4 and 24h post-MCAO.

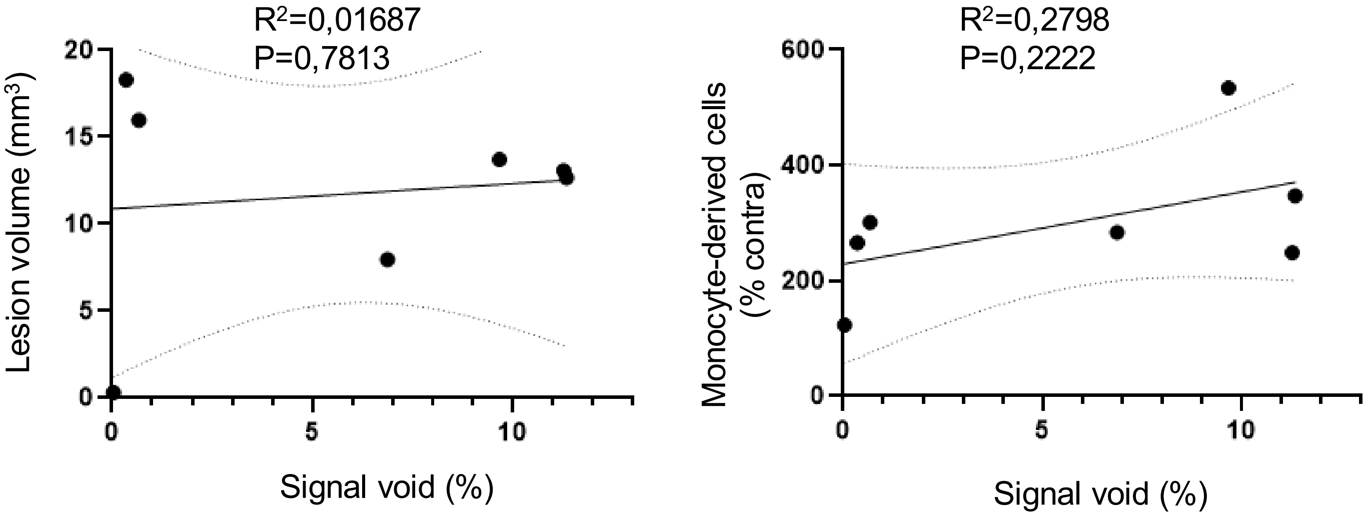

Supplementary Figure S14

Supplementary Figure S14: No correlation between immuno-MRI targeting E-selectin and lesion volume or macrophage infiltration. Assessment of MPIO-αE-selectin-induced signal void versus lesion volume (left) or macrophage infiltration (right). Reported statistics were obtained using Pearson's correlation test.
